# CLIC and membrane wound repair pathways enable pandemic norovirus entry and infection

**DOI:** 10.1038/s41467-023-36398-z

**Published:** 2023-02-28

**Authors:** B. Vijayalakshmi Ayyar, Khalil Ettayebi, Wilhelm Salmen, Umesh C. Karandikar, Frederick H. Neill, Victoria R. Tenge, Sue E. Crawford, Erhard Bieberich, B. V. Venkataram Prasad, Robert L. Atmar, Mary K. Estes

**Affiliations:** 1 Department of Molecular Virology and Microbiology, Baylor College of Medicine, Houston, TX USA; 2 Verna and Marrs McLean Department of Biochemistry and Molecular Biology, Baylor College of Medicine, Houston, TX USA; 3 Department of Physiology, University of Kentucky, Lexington, KY 40506 and VAMC, Lexington, KY 40502 USA; 4 Department of Medicine, Baylor College of Medicine, Houston, TX USA

**Keywords:** Virus-host interactions, Viral pathogenesis, Viral infection

## Abstract

Globally, most cases of gastroenteritis are caused by pandemic GII.4 human norovirus (HuNoV) strains with no approved therapies or vaccines available. The cellular pathways that these strains exploit for cell entry and internalization are unknown. Here, using nontransformed human jejunal enteroids (HIEs) that recapitulate the physiology of the gastrointestinal tract, we show that infectious GII.4 virions and virus-like particles are endocytosed using a unique combination of endosomal acidification-dependent clathrin-independent carriers (CLIC), acid sphingomyelinase (ASM)-mediated lysosomal exocytosis, and membrane wound repair pathways. We found that besides the known interaction of the viral capsid Protruding (P) domain with host glycans, the Shell (S) domain interacts with both galectin-3 (gal-3) and apoptosis-linked gene 2-interacting protein X (ALIX), to orchestrate GII.4 cell entry. Recognition of the viral and cellular determinants regulating HuNoV entry provides insight into the infection process of a non-enveloped virus highlighting unique pathways and targets for developing effective therapeutics.

## Abstract (web summary)

Culture methods for studying human norovirus infection have been recently established. Here, Ayyar and colleagues use human intestinal enteroids to dissect viral entry mechanisms and show that CLIC-mediated endocytosis and wound repair pathways are exploited by pandemic norovirus.

## Introduction

Human noroviruses (HuNoVs), non-enveloped, single-stranded, positive-sense RNA viruses, are classified into at least ten genogroups (GI-GX) and 48 genotypes and belong to the family *Caliciviridae*. HuNoVs are the leading cause of acute gastroenteritis worldwide^1^, posing a significant risk to global health. Specific treatments and vaccines are lacking, partly due to the extended time before a reliable and tractable culture system became available to cultivate HuNoV, which limited understanding of norovirus pathophysiology, drivers of host-virus interactions, and systems to test approaches to control infection.

A breakthrough came when nontransformed human intestinal enteroids (HIEs) were proven to be a reproducible and biologically relevant system that supports the replication of multiple HuNoV strains^2,3^. HIEs have been successfully applied to (1) identify HuNoV strain-specific growth requirements^2,4–6^, (2) identify neutralizing monoclonal antibodies and improve understanding of host responses to HuNoV for vaccine development^7–10^, and (3) monitor the environmental HuNoV load and the efficacy of disinfection strategies^11,12^. HIEs recapitulate the cellular complexity, diversity, and physiology of the human gastrointestinal tract along with host restriction and genetic factors and mimic strain-specific epidemiological host-virus infection patterns, making them an ideal system to dissect HuNoV infection and pathophysiology^13^.

Virus entry into the cell is the first step in infection. It is initiated by interactions between specific motifs on both viral and host surface proteins and activates signaling cascades that destabilize the membrane barrier. Although virus-cell surface interactions are complex and highly variable, the number of pathways utilized by viruses for cell entry is relatively limited based on certain key surface components. Herein, we sought to uncover entry determinants of a globally dominant GII.4 HuNoV strain into physiologically relevant HIEs that support virus replication. Our studies show that GII.4 HuNoV binding to epithelial cells induces plasma membrane wounding that triggers cellular components to the injury site for membrane repair. Surprisingly, we found the virus capsid interacts with the endosomal sorting complexes required for the transport (ESCRT) protein ALIX and induces lysosomal exocytosis along with other membrane repair mechanisms. GII.4 HuNoV enters HIEs using a specific mechanism driven by endosomal acidification and requires effectors of the CLIC pathway [cholesterol, Cdc42, ADP-ribosylation factor 1 (Arf1), and galectin-3 (gal-3)]. These studies demonstrate an active CLIC pathway in HIEs, with a crosstalk between CLIC-mediated endocytosis and host repair mechanisms highlighting these pathways as targets for interfering with pathogen entry in human intestinal cells.

## Results

### GII.4 entry in jejunal HIEs requires endosomal acidification to initiate endocytosis

**Fig. 1:**
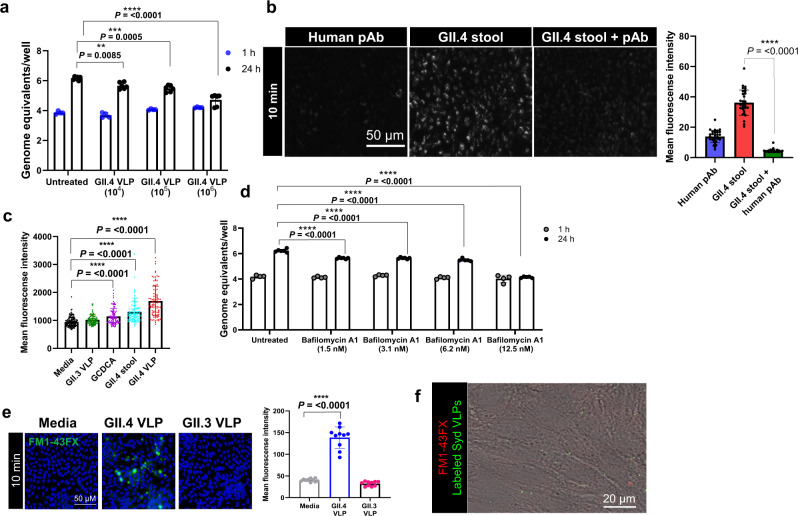
GII.4 capsid protein elicits acidification and endocytosis in HIEs. **a** Viral replication at 24 h (black dots) compared to bound virus at 1 h (blue dots) and inhibition of replication in the presence of VLPs compared to untreated at 24 h. Replication was quantified using *n* = 2 independent HIE replicates for the 1 h and *n* = 3 independent HIE replicates for 24 h with 2 technical replicates/sample. **b** LysoTracker staining of acidic compartments in the presence of an anti-GII.4 polyclonal antibody (pAb), GII.4 virus, and pAb mixed with GII.4 virus at 37 ^o^C. Right panel: Mean fluorescence intensity was quantified from different regions of interest (ROIs) for pAb (blue bar, ROIs = 32), GII.4 stool (red bar, ROIs = 31), GII.4 stool +pAb (green bar, ROIs = 32). **c** LysoTracker staining of acidic compartments induced by GII.3 VLP (green, ROIs = 100), GCDCA (purple, ROIs = 100), GII.4 virus (cyan, ROIs = 101), and GII.4 VLP (red, ROIs = 97) compared to media (black, ROIs = 140). **d** GII.4 replication in the presence/absence of V-ATPase inhibitor bafilomycin A1 at 1 h (bound virus, gray dots) and at 24 h (black dots). Viral GEs were quantified using *n* = 2 independent HIE replicates for the 1 h and *n* = 3 independent HIE replicates for 24 h with 2 technical replicates/sample. **e** FM1-43FX (green) uptake showing GII.4 VLP-induced endocytosis. VLP-induced endocytosis compared to media (*n* = 4 HIE replicates). Right panel: Mean fluorescence intensity quantified from ROI = 10. **f** Time lapse microscopy showing GII.4 VLP (green) endocytosis and FM1-43x uptake (red). All the experiments were repeated independently three times with similar results. In **a**–**e**, error bars represent mean ± SD with significance (*P* values) calculated using one-way ANOVA, Dunnett’s multiple comparisons test. Source data are provided as a Source Data file.

To probe HuNoV entry into a permissive, secretor positive jejunal (J2) HIE line, we used virus-like particles (VLPs) from pandemic GII.4 Sydney/2012 (GII.4 Syd VLPs) and GII.4 Sydney/2012 virus. We first validated VLPs as a surrogate for virus in these studies by showing GII.4 Syd VLPs inhibited virus replication of a GII.4 stool isolate in a dose-dependent manner (Fig. 1a). Several HuNoV strains are dependent on intestinal components such as bile acid (BA) and ceramide for cell entry and replication^6^. GII.4 infection is enhanced by, but does not require, bile or BA for replication^2^, while initiation of endosomal acidification with BA glycochenodeoxycholic acid (GCDCA) is required for GII.3 infection^6^. To understand if endosomal acidification is a requirement for GII.4 entry, HIEs were inoculated with GII.4 virus, and acidic compartments were labeled with LysoTracker. In GII.4-inoculated cells, a significant increase in LysoTracker-positive organelles was seen, which was significantly reduced in the presence of neutralizing GII.4-specific polyclonal antibody (pAb), indicating that acidic organelles are induced in response to virus-cell interactions (Fig. 1b). Endosomal acidification studies showed that GII.4 Syd VLPs, like virus, induced endosomal acidification that was not observed with GII.3 VLPs, indicating strain-specific differences in the ability to trigger endocytosis (Fig. 1c). Moreover, GII.4 infection in the presence of bafilomycin A1, a vacuolar ATPase (V-ATPase) inhibitor, reduced the number of GII.4-induced acidic compartments (Fig. S1a), completely inhibiting GII.4 replication in a dose-dependent manner (Fig. 1d). Time course experiments showed a partial inhibition of GII.4 replication when bafilomycin A1 was present only in the early stages of infection, while complete inhibition was observed when the inhibitor was present throughout the entire infection period [24 h post infection (h)] (Fig. S1b, upper right panel). This was due to the presence of membrane-bound VLPs, which after bafilomycin A1 removal, activated endocytosis reversing inhibition of V-ATPase (Fig. S1b, lower right panel).

GII.4-induced endocytosis was confirmed using FM1-43FX epifluorescence and time lapse microscopy (Fig. 1e, f). FM1-43FX labels extracellular membranes and fluorescent puncta are observed following endocytosis. An upregulation of FM1-43FX-labeled compartments was observed in GII.4 VLP-treated cells further confirming that unlike GII.3 (which requires BA), the GII.4 Syd capsid alone is capable of triggering endocytosis upon interaction with the host cells (Fig. 1e). Endocytosis experiments in the presence of bafilomycin A1 indicated that GII.4 endocytosis is V-ATPase dependent (Fig. S1c) and required the intact VLP as the glycan-binding P domain alone did not trigger endocytosis (Fig. S1d). FM1-43FX labeling experiments in the presence of additional VLPs showed that four GII.4 variants [Houston Virus/2002 (HOV), New Orleans/2009 (NO), Grimsby/1995 (GRV), Farmington Hills/2002 (FH)] induced endocytosis while HuNoV strains that require BA (GII.3, GI.1 and GII.17) did not induce endocytosis, further supporting the use of different cell entry mechanisms between BA-dependent and independent HuNoV strains (Fig. S1d).

### GII.4 uses a dynamin-independent pathway that requires membrane cholesterol and is influenced by actin

**Fig. 2:**
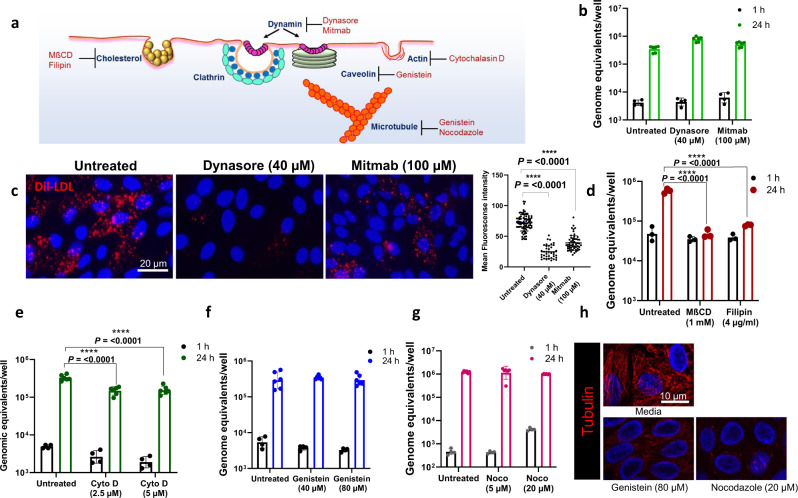
GII.4 entry is dynamin-independent but depends on cholesterol and actin for infection. **a** Schematic of major endocytosis pathways, key regulators and their inhibitors. **b** GII.4 replication (at 37 ^o^C) was assessed in the presence of dynamin inhibitors, dynasore and mitmab. Viral RNA replication was quantified at 1 (black) and 24 h (green) by RT-qPCR. **c** Validation of dynamin inhibitor activity by Dil-complexed low density lipoprotein (Dil-LDL) uptake (red). Right panel: Quantitation of Dil-LDL uptake in untreated (80 ROI), dynasore-treated (38 ROI) and mitmab-treated (55 ROI) HIEs. **d** GII.4 replication in the presence of cholesterol sequestrants, MßCD and filipin, at 1 h (black) and 24 h (red) at 37 ^o^C. **e** GII.4 replication in the presence of actin depolymerizing agent cytochalasin D (Cyto D) at 1 h (black) and 24 h (green). **f** GII.4 replication in the presence of receptor tyrosine kinase (RTK) inhibitor genistein at 1 h (black) and 24 h (blue) at 37 ^o^C. **g** GII.4 replication in the presence of nocodazole, a microtubule disrupting agent, at 1 h (black) and 24 h (pink) at 37 ^o^C. **h** Tubulin staining (red) in the presence of genistein and nocodazole. All the experiments were repeated independently three times with similar results. In **b**, **d**–**g** viral GEs were quantified using *n* = 2 independent HIE replicates for the 1 h and *n* = 3 independent HIE replicates for 24 h with two technical replicates/sample. In **b**, viral GEs were quantified using *n* = 3 HIE replicates. In **b**–**g**, error bars represent mean ± SD with significance relative to untreated control calculated using one-way ANOVA, Dunnett’s multiple comparisons test. Source data are provided as a Source Data file.

GII.4 entry was explored further by using specific inhibitors of membrane components known to be involved in endocytic pathways (Fig. 2a). Dynamin, important for both clathrin- and caveolin-mediated endocytosis, was first tested considering the entry of murine norovirus (MNV) in RAW264.7 macrophages is dynamin-dependent^14^. Inhibition of dynamin with specific inhibitors, dynasore and mitmab, did not alter GII.4 infection, showing that clathrin- and caveolin-mediated endocytosis pathways are not involved in GII.4 cell entry (Fig. 2b). The efficacy of dynasore and mitmab in HIEs was confirmed using a fluorescently labeled, low-density lipoprotein (LDL)-uptake assay, where both inhibitors reduced LDL uptake (Fig. 2c) at the tested concentrations (40 and 100 µM).

We next targeted cholesterol, another abundant plasma membrane component implicated in the entry and replication of many viruses, including GII.3 HuNoV^6,14–18^. Cholesterol depletion in HIEs, by inhibitors methyl-β-cyclodextrin (MβCD) and filipin, reduced GII.4 infection significantly (Fig. 2d, Table S1). In addition, a lower level of inhibition in GII.4 replication with U18666A, an intracellular cholesterol transport inhibitor, was observed (Fig. S2a) compared to MβCD and filipin. The activity of U18666A in HIEs was confirmed using filipin and CD63 staining, indicating defective intracellular cholesterol transport (Fig. S2b). Altogether, these results show that cholesterol is critical for GII.4 replication.

We next evaluated GII.4 entry dependence on cytoskeletal components using inhibitors against actin, receptor tyrosine kinases (RTK), and microtubules. Cytochalasin D, an actin-depolymerizing agent, reduced GII.4 replication significantly (53%) (Fig. 2e), whereas genistein (RTK and caveolin inhibitor) and nocodazole (microtubule inhibitor) had no effect on GII.4 replication (Fig. 2f, g). Tubulin staining confirmed the activities of genistein and nocodazole in HIEs (Fig. 2h), suggesting that actin, but not microtubule or RTKs, is essential for GII.4 entry.

### GII.4 depends on the CLIC pathway for cell entry

**Fig. 3:**
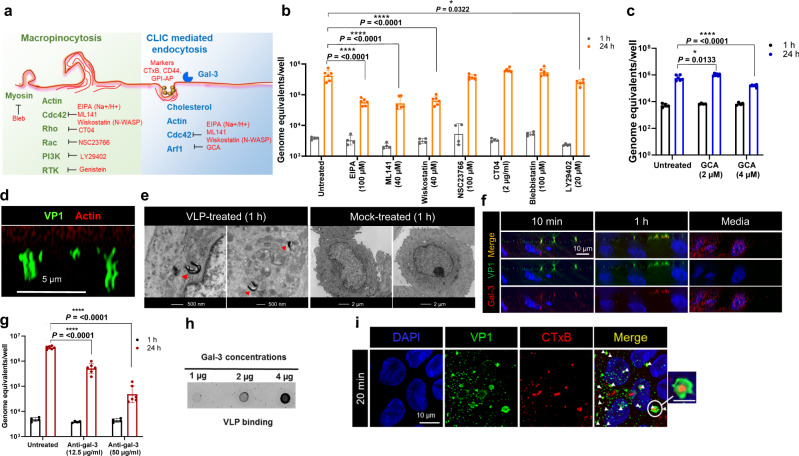
GII.4 infection in HIEs is sensitive to effectors of CLIC pathway. **a** Schematic of macropinocytosis and the Clathrin Independent Carrier (CLIC) pathways, their effectors and inhibitors. **b** GII.4 replication in the presence of EIPA (Na^+^/H^+^ exchanger inhibitor), ML141 (Cdc42 inhibitor), Wiskostatin (N-WASP inhibitor), NSC23766 (RAC1 inhibitor), CT04 (RhoI inhibitor), Blebbistatin (myosin inhibitor), and LY29402 (PI3K inhibitor) at 1 h (gray) and 24 h (orange). **c** GII.4 replication in the presence of Arf1 inhibitor Golgicide A (GCA at 1 h (black) and 24 h (blue). **d** GII.4-induced tubular carriers at 1 h (37 ^o^C) detected using guinea pig anti-Sydney VLP polyclonal antibody (Gp Syd-pAb) for viral capsid (green) and phalloidin for actin (red). Images were taken using a ZEISS Laser Scanning Microscope LSM 980 with Airyscan 2. **e** Electron microscopy to identify CLIC structures in GII.4 VLP-treated HIEs. (1–7 structures/cell) compared to, mock-treated cells (no structures in *n* = 25 cells). **f** Confocal microscopy to detect GII.4 VP1 capsid (green) colocalization with gal-3 (red) on the cell surface at 10 min and 1 h (37 ^o^C) after VLP treatment using anti-gal-3 and Gp Syd-pAb (n = 3 HIE replicates). **g** Effect of blocking GII.4 virus-galectin-3 (gal-3) surface interaction using anti-gal-3 antibody on GII.4 replication at 1 h (black) and 24 h (red). **h** Dot blot analysis investigating GII.4 VLP interaction with purified gal-3. **i** Probing CLIC carriers utilized in HIEs for endocytosis with Alexa Fluor™ 594 conjugated cholera toxin B (CTxB) (red) and GII.4 VLPs (green) with similar cargoes marked by white arrows (*n* = 2 HIE replicates). Inset: Co-occurrence of CTxB and GII.4 VLPs in similar cargos, scale = 5 µm. All the experiments were repeated independently three times with similar results. In **b**, **c**, and **g** viral GEs were quantified using *n* = 2 independent HIE replicates for the 1 h and *n* = 3 independent HIE replicates for 24 h with two technical replicates/sample. The error bars represent mean ± SD with *P* values calculated using one-way ANOVA, Dunnett’s multiple comparisons test with comparisons at 24 h relative to untreated control. Source data are provided as a Source Data file.

Inhibition of GII.4 entry by cytochalasin D showed that viral entry was influenced by actin manipulation, but this drug was cytotoxic above the concentrations tested. This led us to examine other regulators of actin mechanics that influence clathrin-independent endocytosis (CIE). After confirming that the concentration of inhibitors used in assays did not induce cytotoxicity (Table S1), we tested macropinocytosis and CLIC endocytosis (Fig. 3a), two major CIE pathways, focusing on Cdc42 [EIPA, ML141, wiskostatin (Wisko)], Rac1 [NSC23766 (NSC)], and RhoA (CT04). Inhibition of Cdc42 (EIPA, ML141, and Wisko) dramatically reduced GII.4 replication (86–90%) (Figs. 3b and S3a), whereas inhibiting Rac1 or RhoA had no effect on GII.4 replication. Other inhibitors of classical macropinocytosis such as smooth muscle myosin II inhibitor [blebbistatin (Bleb)] and PI3K inhibitor [LY29402 (LY)], known to efficiently block the entry of vaccinia virus^19^, human adenovirus type 3^20^, and respiratory syncytial virus^21^, were also tested along with inhibitors of epidermal growth factor receptor (EGFR) (CAS879127-07-8) and protein kinase C (PKC) (calphostin). None of these inhibitors of CIE had any effect on GII.4 replication (Figs. 3b and S3b), emphasizing that GII.4 entry is via a Cdc42-specific mechanism distinct from a classical macropinocytosis pathway. The efficacy of inhibitors in HIEs was confirmed by actin staining, which detected morphological changes in treated cells (Fig. S3c). Next, we evaluated GII.4 infection in the presence of an Arf1 inhibitor, Golgicide A (GCA), and found that GCA significantly inhibited GII.4 replication (65–75%) at 24 h (Fig. 3c).

Taken together, the inhibitor data suggest that GII.4 entry relies on cholesterol, Cdc42 and Arf1, indicating a CLIC-mediated virus entry pathway. However, inhibitors can have many off-target effects, so we used a multipronged approach by evaluating the morphology of CLIC carriers, assessing GII.4 association with gal-3 (a regulator of the CLIC pathway)^22^ and testing GII.4 colocalization with known markers of the CLIC pathway. Fluorescence microscopy showed that GII.4 induces tubular structure formation reminiscent of CLIC carriers in VLP-treated cells with variable lengths (200 nm–2 µm) (Fig. 3d), which were further confirmed by electron microscopy (EM) (Fig. 3e). EM also showed VLPs in vacuolar structures similar to those observed in mouse jejunal enterocytes (Fig. S3d) ^23^. Next, GII.4 association with gal-3 was assessed using fluorescence microscopy, gal-3 inhibition assays, and direct binding respectively (Figs. 3f, S3e, S3f, 3g, 3h, and 3i). We found that GII.4 colocalizes with gal-3 (Fig. 3f) in a time-dependent manner. In the presence of TD-139, a specific gal-3 inhibitor, both GII.4 colocalization and endocytosis is rapidly (10 min) inhibited suggesting the importance of gal-3 in GII.4 entry into cells (Figs. S3e and S3f). Gal-3 is a multifunctional protein involved in many cellular processes and is found in both the biotinylated surface protein (BSP) plasma membrane and cytosolic fractions of cells (Fig. S3g). As inhibitors are unable to differentiate between the different populations of gal-3, we specifically used anti-gal-3 antibody to target gal-3 interactions on the plasma membrane. Our results showed that anti-gal-3 antibody significantly inhibited GII.4 replication (98% reduction), further asserting the importance of gal-3-GII.4 interaction in GII.4 entry and infection (Fig. 3g). Direct binding assays showed that gal-3 bound to VLPs (Fig. 3h), and to the GII.4 capsid S domain, but not to the P domain, of VP1 of VLPs, with micromolar affinity (Figs. S3h and S3i).

Finally, we investigated GII.4 colocalization using specific markers of the CLIC pathway such as cholera toxin B (CTxB), CD44 and glycosylphosphatidylinositol-anchored protein (GPI-AP). All these proteins are well studied and characterized for their usage in CLIC-mediated endocytosis^24–26^. Fluorescence microscopy showed that GII.4 shared cargoes with CTxB (Fig. 3i) despite not having any effect on replication when recombinant CTxB was used as a competitor (Fig. S3j). This suggests that although GII.4 uses a similar entry pathway as CTxB, the receptor/s used for each is distinct. GII.4 colocalization with CD44 and GPI-AP further verified that GII.4 uses CLIC-mediated endocytosis for entry (Figs. S3k and S3l).

### ESCRT protein ALIX is critical for GII.4 entry

**Fig. 4:**
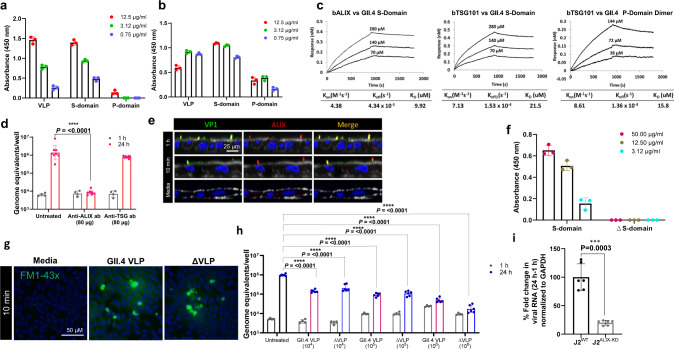
GII.4 interacts with ALIX and TSG101, with ALIX being critical for virus entry. **a** GII.4 VLP, S and P domain binding to immobilized ALIX by ELISA. Error bars indicate mean ± SD with three replicates. **b** GII.4 VLP, S and P domain binding to immobilized TSG101 by ELISA. Error bars indicate mean ± SD with three replicates. **c** Bio-Layer Interferometry (BLI) to determine the binding affinity of GII.4 S and P domains to biotinylated ALIX and TSG101 (bALIX and bTSG101). **d** Effect of blocking virus interaction with ALIX and TSG101 on viral replication using specific pAbs at 1 h (gray) and 24 h (pink). Error bars indicate mean ± SD calculated using 2 HIE replicates for 1 h and 4 HIE replicates for 24 h (with two technical replicates/sample). **e** Confocal microscopy to probe GII.4-ALIX colocalization on the cell surface by detecting VP1 (green) and ALIX (red) using Gp Syd-pAb and rabbit anti-ALIX pAb. **f** ELISA to evaluate binding of GII.4 S- and mutant ∆S-domains to immobilized ALIX. Error bars indicate mean ± SD with 3 replicates. **g** Endocytosis induced by wild type GII.4 VLP and GII.4 VLP lacking the ALIX–binding motif (Δ VLP) using FM1-43FX in HIEs at 37 ^o^C (*n* = 3 HIE replicates). **h** Viral replication in the presence of GII.4 WT VLPs (pink bars) and Δ VLPs (blue bars) was compared to untreated (black bar) at 24 h (blue dots). 1 h (gray dots) represents bound virus. Error bars indicate mean ± SD calculated using 2 HIE replicates for 1 h and 3 HIE replicates for 24 h (each condition with two technical replicates). **i** GII.4 replication in WT J2 and J2^ALIX-KD^ HIE monolayers indicated by percent fold change in viral RNA using the 2 − ΔΔCT method. Error bars indicate mean ± SD calculated using 3 HIE replicates for each condition with two technical replicates. All the experiments were repeated independently three times with similar results. *P* values for **d**, **h**, and **i**, relative to untreated control were calculated using one-way ANOVA, Dunnett’s multiple comparisons test. Source data are provided as a Source Data file.

ESCRTs are known regulators of endocytosis. The unexpected presence of conserved ESCRT binding motifs for ALIX and TSG101 in the GII.4 capsid sequence (Fig. S4a) led us to further investigate if these proteins are involved in GII.4 entry. Binding assays (ELISA and dot-blot) showed that GII.4 Syd VLPs interact with recombinant ALIX and TSG101 (Figs. 4a, b, S4b). Confocal microscopy of virus-infected HIEs showed colocalization between replicating virus, and ALIX and TSG101 at 24 h, suggesting their role in GII.4 replication (Fig. S4c). Putative ESCRT binding sequences are present in both the GII.4 capsid S and P domains (Fig. S4a); however, ELISA data showed that while TSG101 binds to both the S and P domains (Fig. 4b), ALIX binding is limited to the S domain only (Fig. 4a). Bio-Layer Interferometry (BLI) analysis confirmed the ELISA results, demonstrating that binding affinity of ALIX to the S domain (*K*_*D*_ = 9.92 µM) and TSG101 to the S domain (*K*_*D*_ = 21.5 µM) and the P domain (*K*_*D*_ = 15.5 µM) were in the micromolar range (Fig. 4c).

We further probed the contribution of ALIX and TSG101 in GII.4 entry using protein-specific antibodies as blocking agents. While anti-ALIX pAb blocked GII.4 replication completely in a dose-dependent manner, anti-TSG101 pAb had no effect on GII.4 replication (Figs. 4d and S4d). These data suggest that ALIX is accessible to GII.4 VLPs on the membrane surface, which was further confirmed by Western blot detection of ALIX in the biotinylated surface protein (BSP) fraction of HIEs with an anti-ALIX pAb (Fig. S4e). Confocal microscopy established a direct link between GII.4 and ALIX as HIEs incubated with VLPs (10 min or 1 h) showed GII.4 colocalization with ALIX at both time points, with more VLP-ALIX colocalization observed at 1 h compared to 10 min (Fig. 4e). Notably, media-treated control cells exhibited no significant cell surface ALIX staining compared to VLP-treated cells, implying that GII.4 binding induced ALIX recruitment to the cell surface (Fig. 4e).

Both ELISA and BLI experiments showed that ALIX binds the GII.4 capsid S-domain, with a conserved “LYTPL” sequence (Fig. S4a). This sequence was mutated (∆) to “AATAL” and this ∆S-domain was expressed and purified from *E. coli*. ELISA showed that the ∆S-domain does not bind ALIX, thus, confirming the ALIX binding site (Fig. 4f). To further understand the role of ALIX in entry, an ALIX mutant VLP (∆VLP) was generated by substituting the “LYTPL” sequence with “AATAL” in GII.4 Syd VLP. After confirming that the ALIX mutation did not interfere with VLP formation (Fig. S4f), GII.4 Syd VLP and ∆VLP were analyzed using both endocytosis and viral inhibition assays. Both assays showed that the ∆VLP was identical to GII.4 Syd VLP in terms of inducing endocytosis and the ability to block GII.4 viral replication (Figs. 4g and 4h), which led us to reassess ∆VLP binding to ALIX. Binding assays showed that although ∆VLP (unlike the ∆S-domain) bound to ALIX, the binding was reduced compared to the GII.4 Syd VLP (Figs. S4g and S4h). This suggests that, like TSG101, there may be more than one ALIX binding site present on the viral capsid in addition to the site present in the S-domain. To study the role of ALIX in GII.4 infection, attempts were made to generate a J2 ALIX knock-out HIE but were unsuccessful because the KO cultures did not survive. This led us to generate J2 ALIX knock-down (J2^ALIX-KD^) HIEs expressing an ALIX-shRNA and a TurboGFP sensor (Fig. S4i). The J2^ALIX-KD^ cells demonstrated a significant reduction in ALIX expression (75% reduction) compared to the parental J2 cultures (Fig. S4j) and in GII.4 replication (75% reduction) (Fig. 4i) indicating an active role of ALIX in GII.4 infection.

### Endolysosomal dynamics and membrane repair mechanisms regulate GII.4 entry

**Fig. 5:**
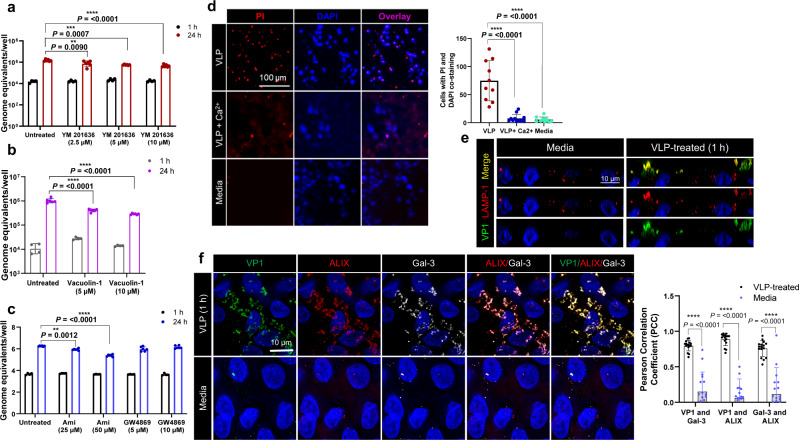
GII.4 entry is sensitive to factors controlling endo-lysosomal homeostasis and induces membrane wounding and subsequent wound repair mechanisms. **a** GII.4 replication in the presence of PIKfyve inhibitor YM201636 at 1 h (black) and 24 h (red) at 37 ^o^C. **b** GII.4 replication in the presence of vacuolin-1 (a lysosomal exocytosis inhibitor) at 1 h (gray) and 24 h (purple). **c** GII.4 replication in the presence of acid sphingomyelinase (ASM, amitriptyline) and neutral sphingomyelinase (NSM, GW4869) inhibitors at 1 h (black) and 24 h (blue). **d** Cell injury determination using propidium iodide (PI) uptake assay. Right panel: Graph quantitating membrane injury calculated by counting number of PI spots (red) counterstained with DAPI (blue) when HIEs were incubated with VLPs (ROI = 10), VLP + Ca^2+^ (ROI = 12) and media (ROI = 10). Significance was calculated using two-way ANOVA, Tukey’s multiple comparisons test. **e** Immunofluorescence staining showing GII.4-induced lysosomal exocytosis represented by the presence of LAMP-1 on the apical cell surface in media-treated cells compared to VLP-treated cells. LAMP-1 (red) and VP1 (green) colocalization was detected using mouse anti-LAMP-1 mAb and Gp Syd-pAb. (*n* = 3 HIE replicates). **f** VP1, ALIX and Gal-3 colocalization in VLP-treated and media-treated cells (1 h at 37^o^C) using confocal microscopy. VP1 (green), ALIX (red) and gal-3 (white) were detected using Gp Syd-pAb, rabbit anti-ALIX pAb and rat-anti-gal3. Right panel: Graph showing colocalization between VP1, gal-3 and ALIX as estimated by Pearson Correlation Coefficient using EzColocalization (ROI = 16, black for VLP- and ROI = 17, blue for media-treated HIEs). Error bars indicate mean ± SD and *P* values were calculated using two-way ANOVA, Šídák’s multiple comparisons test. All the experiments were repeated independently three times with similar results. Error bars for **a**–**c** indicate mean ± SD calculated using 2 HIE replicates for 1 h and 3 HIE replicates for 24 h with two technical replicates/sample. *P* values were calculated using one-way ANOVA, Dunnett’s multiple comparisons test by comparing replication at 24 h to untreated control. Source data are provided as a Source Data file.

Endolysosomal dynamics play an important role in GII.3 replication^6^, which led us to investigate the same for GII.4. This was achieved by disrupting endosomal maturation and trafficking using YM201636 [FYVE-type zinc finger-containing phosphoinositide kinase (PIKfyve) inhibitor] and vacuolin 1 (PIKfyve and lysosomal exocytosis inhibitor), which resulted in up to 70–80% reduction in GII.4 replication compared to untreated cells (Fig. 5a, b). Time course experiments showed the presence of inhibitors up to 1 h reduced virus yield by 40%, whereas up to 80% inhibition in yield was observed when the inhibitors were present up to 2 h or 24 h, thus, suggesting the inhibitor was effective in the early stages of GII.4 entry (Figs. S5a and S5b).

Inhibition of lysosomal exocytosis by vacuolin-1 led to a reduction in GII.4 replication, indicating that GII.4 might require acid sphingomyelinase (ASM) translocation and surface ceramides for infection like other caliciviruses^6,18^. Therefore, we assayed different enzymes involved in ceramide synthesis for their effect on GII.4 replication using inhibitors against ASM (amitriptyline), neutral sphingomyelinase (NSM; GW4869), and ceramide synthase [fumonisin 1 (FB1)]. In addition, we tested if addition of exogenous sphingomyelin (SM), a lipid product upstream of ceramide, has any effect on GII.4 replication. Among the inhibitors tested, amitriptyline significantly inhibited GII.4 replication (up to 85%) whereas GW4869, SM addition and FB1, had no effect confirming that like GII.3, GII.4 requires ASM activity (Figs. 5c, S5c and S5d). FB1-treated cells had reduced CD95 expression and enhanced intracellular lysosomal associated membrane protein 1 (LAMP-1) expression compared to untreated cells, which confirmed the activity of FB1 in HIEs (Fig. S5e). Since GII.4 does not require BA for infection, we postulated that GII.4-induced lysosomal exocytosis may catalyze ceramide generation as reported with feline calicivirus (FCV) or MNV in porcine kidney LLC-PK cells^18^. Using a ceramide-specific rabbit antibody^27^, we established that GII.4 VLPs induce surface ceramides in HIEs by confocal microscopy (Fig. S5f), confirming that ASM translocation and ceramide generation are associated with GII.4 infection.

Lastly, we examined cellular mechanisms associated with lysosomal exocytosis, ASM translocation and surface ceramide generation, which are known to be associated with endocytosis and wound repair mechanisms^28^. GII.4 association with membrane wounding was investigated using a propidium iodide (PI) uptake assay. PI uptake was significantly increased in the presence of GII.4 Syd VLPs (10 min incubation) compared to untreated cells and GII.4 Syd VLP-treated cells with calcium, which facilitates membrane repair by early signaling^29^ (Fig. 5d). GII.4 induction of membrane wounding was associated with VLP binding to cells at 4 ^o^C (Fig. S5g) and requires initial VLP interaction with cell surface histo-blood group antigens (HBGA), glycans known to be essential for virus infection. Thus VLP-induced membrane wounding did not occur in isogenic HIEs in which the *fucosyltransferase 2* (*FUT2*) gene was knocked out and thus do not express secretor-dependent HBGAs on their plasma membranes^4^ (Fig. S5h). GII.4-induced membrane wounding was further confirmed by staining the VLP-treated and untreated cells for (1) LAMP-1 and (2) regulators of endo-lysosomal processes and wound healing mechanisms, Rab11 and Rab14^30,31^. GII.4 VP1 localized with LAMP-1 (Fig. 5e), Rab11 (Fig. S5i), and Rab14 (Fig. S5j), on the plasma membrane of HIEs treated with GII.4 Syd VLPs, validating GII.4 entry is associated with membrane wounding and healing mechanisms. Subsequently, we revisited gal-3 and ALIX, as both are known to be coordinating factors in detecting membrane damage and repair^32,33^. As expected, we saw that trafficking of both gal-3 and ALIX to the cell surface was induced by GII.4 Syd VLP-treatment compared to untreated cells and a high degree of colocalization was observed between gal-3, ALIX, and GII.4 VP1 (Fig. 5f), consistent with the fact that GII.4 enters the cell by compromising membrane integrity.

## Discussion

Viruses exploit many cellular pathways for cell entry. Sometimes similar effectors influence multiple endocytosis routes making it challenging to pinpoint a specific entry mechanism^34^. For example, both porcine sapovirus (PoSaV) and feline calicivirus (FCV) are endocytosed via dynamin- and cholesterol-dependent clathrin-mediated endocytosis into kidney cells^35,36^, whereas murine norovirus (MNV) is endocytosed via dynamin- and cholesterol-dependent, clathrin- and caveoli-independent endocytosis into RAW264.7 macrophages^14^. Since most studies of virus entry are carried out in immortalized cell lines that do not fully recapitulate the properties of the original host, characterization of virus entry into HIEs offers previously unrealized insights into intestinal biology^37^. HIEs are relevant in that they are multicellular, nontransformed, physiologically active human cultures that contain the necessary susceptibility factors for human tropic HuNoV infection.

**Fig. 6:**
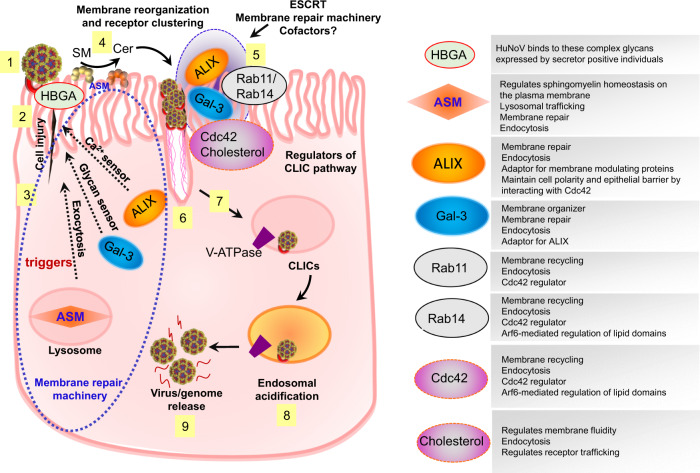
GII.4 uses a complex entry mechanism involving cellular wound repair mechanisms and CLIC-mediated endocytosis. (1) Binding of GII.4 with its glycan receptor (HBGAs and possibly with a still unidentified co-receptor) on the cell surface (2) induces plasma membrane wounding (3) triggering signaling responses that direct multiple membrane repair cellular components to the injury site. (4) ASM translocation to the plasma membrane surface results in conversion of sphingomyelin (SM) to ceramide. (5) Ceramide formation along with other membrane repair processes involving gal-3 (glycan damage sensor), ALIX (Ca^2+^ sensor) and membrane recycling processes regulated by Rab11 and Rab14 result in membrane reorganization and receptor clustering leading to (6) tubular carrier formation due to multiple interactions of virus with host factors causing membrane bending and (7) endocytosis regulated by Cdc42 and cholesterol. (8) GII.4 entry into the cell results in V-ATPase regulated endosomal acidification causing (9) conformational changes in the virus capsid and release of the viral genome from the endosomal compartment.

Here, we report the pandemic GII.4 virus and VLPs initiate a previously uncharacterized and complex entry process into HIEs that involves carriers of a dynamin-independent, clathrin-independent endocytosis pathway, gal-3, the ESCRT protein ALIX, and wound repair mechanisms (Fig. 6). To our knowledge, this viral entry process into nontransformed HIE cells was not previously described, although individual components have been associated with entry of several other viruses into a range of different cell types.

VLPs were used for many of our entry studies because infectious virus cannot yet be passaged indefinitely and current yields of virus from infected HIEs are not sufficient to produce purified particles for extensive biochemical studies. Since 1992, when HuNoV VLPs were first produced and characterized^38^, they have been extensively documented as useful surrogates for studies of virus structure^39–42^, antigenicity, immunogenicity, and glycan binding^43–45^. Here, we validated that VLPs are useful for studying virus entry by documenting their effectiveness as competitors of homologous virus replication. VLPs also recapitulate virus differences as VLPs of different strains exhibit differences in virus entry (Fig. S1d). GII.4 is recognized to bind to HBGA glycans through the P domain of the capsid^46,47^, yet surprisingly, endocytosis into HIEs was not observed with the P domain (dimer) alone but was observed only with complete VLPs (having 180 copies of the capsid VP1 protein). These results indicate that the capsid shell domain participates in virus entry into cells and add evidence to the hypothesis that cells contain an entry co-receptor in addition to HBGA glycans that serve as initial binding factors for HuNoV infection. Alternatively, it could be that avidity effects, such as multiple interactions linked together, are important for endocytosis.

We show that GII.4 uses the CLIC pathway for entry in a HBGA glycan-dependent manner (Fig. 6). The CLIC pathway is reported to be involved in uptake of abundant surface proteins such as CD44 and glycosylphosphatidylinositol-anchored proteins^25,26^, bacterial toxins (CTxB and Shiga)^24,48^, adeno-associated virus^49^ and SARS-CoV-2^50^. Use of the CLIC pathway by GII.4 HuNoV in nontransformed HIEs is consistent with the CLIC pathway being operational in vivo in mouse enterocytes^23^ and tubular invaginations observed in giant unilamellar vesicles treated with GII.4 VLPs^51^. Our study shows that gal-3 is recruited by GII.4 at the site of the GII.4-membrane interface and GII.4-gal-3 interactions are necessary for infection, suggesting a putative role of gal-3 in endocytosis. Although intracellular gal-3 and extracellular gal-3 have a myriad of functions, extracellular gal-3 is a key player in the biogenesis of CLIC structures^22,23^. Gal-3 is also known for its crosslinking abilities, organizing membrane proteins/receptors and influencing their function and signaling^52^. We hypothesize that, as reported with CD44, monomeric gal-3 interacts with GII.4 (bound to its still uncharacterized receptor) on the membrane surface leading to oligomerization of the receptor-GII.4-gal-3 complex, and interaction of this complex with multiple glycosphingolipid head groups in the outer plasma membrane leaflet causes membrane bending due to an avidity effect^22^ and subsequently leads to endocytosis. Although GII.4 VLPs are shown to be intrinsically capable of forming tubular structures in giant unilamellar vesicles, it is unknown if the formation of such structures alone is sufficient for endocytosis. Moreover, the GII.4-cell interaction causes recruitment of ALIX, an essential component of CLIC-mediated endocytosis^53^ and an adaptor for several ESCRT-III subunits and phospholipids capable of regulating membrane curvature and tubular structure formation^54–56^, consistent with the notion that GII.4 triggers signaling pathways that accrue an ensemble of cellular factors with membrane modulating functions for cell entry.

This leads to many interesting questions such as how does GII.4 engage gal-3 and ALIX, what prompts their recruitment to the membrane surface to induce endocytosis and is there an association between gal-3 and ALIX? Interaction of gal-3 and ALIX with the normally inaccessible S domain of the GII.4 capsid suggests that the GII.4 capsid undergoes conformational changes, which can be attributed to the newly recognized plasticity of VLPs under certain conditions^42,57^. These conformational changes in the GII.4 capsid may be due to its binding to the initial glycan receptor through the P domain, thus, exposing the S domain to participate in virus-host interaction. This suggests that the GII.4 P domain interaction with the glycan receptor may be instrumental in triggering gal-3 and ALIX recruitment to the cell surface, which is further supported by the fact that we see increased gal-3 and ALIX recruitment with increased VLP binding. However, the GII.4 S domain interaction is still required with gal-3 and ALIX to drive receptor oligomerization and subsequent endocytosis, which is not possible with P domain alone.

To understand GII.4-triggered gal-3 and ALIX translocation to the membrane, we investigated both endolysosomal trafficking and membrane wounding mechanisms due to the well-documented role of gal-3 and ALIX in both processes^32,33^. Wounding experiments demonstrated that GII.4 particles directly induce membrane wounding, which requires plasma membrane glycan expression (Figure S5h) and leads to lysosomal exocytosis and generation of surface ceramides (Figs. 5e and S5f). These events indicate that GII.4 exploits membrane wounding and subsequent repair mechanisms, similar to non-enveloped adenovirus^58^ and the protozoan parasite *Trypanosoma cruzi* in HeLa cells^59^, for infection.

Membrane wounds are repaired by reducing membrane tension, lesion removal by endocytosis, or shedding of wounded areas^28,29,60^, and compelling evidence implicate the involvement of the gal-3, ALIX and ESCRT proteins in membrane repair mechanisms^32,33,60^. Gal-3 is reported to recruit ALIX in response to lysosomal damage, further promoting interaction between ALIX and the ESCRT III effector CHMP4^33^, which is known for its membrane-modulating functions. Moreover, ALIX is known to regulate epithelial cell polarity and maintain the integrity of the epithelial barrier by physically interacting with F-actin by binding the Rho family of small GTPases Cdc42 and Rac1^61^, responsible for actin polymerization and endocytosis. Cdc42-induced cell polarization is regulated by membrane recycling pathways^62,63^ (involving Rab11 and Rab14), which are critical for both wound healing and CLIC-mediated endocytosis. This indicates that GII.4-cell interactions trigger a series of signaling events and cross talk between cellular components activating membrane repair and recycling events that culminates in endocytosis. It is known that inhibiting regulators of the CLIC pathway deters membrane repair processes^64,65^, further validating our results on GII.4 entry, where inhibiting ALIX, lysosomal exocytosis, and ASM translocation inhibited GII.4 replication. Although, the role of ASM or ceramide in the CLIC pathway is unclear, considering the fact that ASM mediates trafficking of palmitoylated proteins to the plasma membrane (influencing lipid raft formation)^66^ and ceramide production leads to plasma membrane repair via endocytosis^28^, both ASM and ASM-generated ceramide seem to be essential for GII.4 entry and infection. Our observation that endocytosis is not observed by adding ceramide exogenously^6^, confirms that endocytosis requires both ASM translocation and subsequent conversion of membrane sphingomyelin to ceramide.

Ceramides and sphingolipids play multifaceted roles as mediators of membrane dynamics by influencing cellular signaling, modulating receptor conformation, and clustering, which can critically affect endocytosis^67,68^. GII.4 binds HBGAs in glycosphingolipid-containing membranes similar to gal-3, which is a glycan sensor and has a high affinity for HBGAs^69,70^. Our data in isogenic HIEs lacking FUT2-dependent HBGAs on the cell surface show that GII.4 binding to such HBGAs is necessary to induce membrane wounding. We posit this triggers ceramide-mediated signaling and changes in membrane/receptor dynamics leading to gal-3 recruitment to the interaction site and promoting cross-talk between GII.4 and effectors of membrane repair and recycling. We postulate that recruited gal-3 acts both as crosslinking membrane organizers and as a switch in immobilizing the ESCRT machinery followed by subsequent endocytosis and scission of CLIC structures. Although gal-3 is a multifunctional protein strictly regulated by its ability to recognize exposed glycans, the exact mechanism of how it fits into the cell signaling process and recruits ESCRT proteins remains unclear and requires further investigation.

In this manuscript, we have shown that GII.4 HuNoV exploits unexpected entry mechanisms to enter the cell by causing membrane wounding, triggering membrane repair mechanisms and inducing endocytosis and endosomal acidification for viral infection (Fig. 6). Our data clearly highlight the strain-specific similarities and differences between non-pandemic, BA-dependent HuNoV strains and the pandemic, BA-independent GII.4 HuNoV. Based on our results, we posit that the dominance of GII.4 over other HuNoV strains may be attributed to its superior ability to exploit constitutive endocytic pathways reserved for recycling abundant surface proteins and membrane repair, in addition to its ability to overcome the host innate response^5^. Other highly pathogenic microbes and toxins (SARS-CoV-2, Shiga toxin and CTxB) share many common features of entry into cells with GII.4 HuNoV entry mechanisms described herein^24,48,50^. Further identification of a specific receptor coupled with a detailed understanding of the role of each of the cellular factors identified here for infection will be helpful in deciphering the specific cellular tropism of GII.4 HuNoVs. This raises interesting questions regarding whether utilization of a particular endocytic pathway by these highly infectious pathogens is associated with their pathogenesis and whether features of the CLIC route of entry and associated membrane repair mechanisms represent new therapeutic targets.

## Methods

### Virus and VLPs

The virus used in these studies was TCH12-580, a GII/Hu/US/2012/GII.4 Sydney [P31]/TCH 12–580 strain. The stool sample was prepared as 10% stool filtrates in phosphate-buffered saline (PBS) with viral titer determined by real-time RT-PCR^2^. VLPs were produced in a baculovirus expression system using open reading frame 2 (ORF2) + ORF3+ untranslated region (UTR) sequences representing HuNoV strains (Table 1).

**Table 1:** Norovirus VLP specifications

Genotype	Variant	GenBank Accession No.
GII.4	Sydney (Syd)	JX459908
GII.4	Grimsby (GRV)	AJ004864
GII.4	Lanzou (HoV)	EU310927
GII.4	Farmington Hills (FH)	AY502023
GII.4	New Orleans (NO)	GU445325
GII.4	Sydney Alix mutant	NA
GII.3	TCH04-577	AB365435
GI.1	Norwalk	M87661 https://www.ncbi.nlm.nih.gov/nuccore/106043086/
GII.17	Katrina	ABD95934

### Pharmacological inhibitors, additives, recombinant proteins, and antibodies

A list of the various inhibitors, additives and recombinant proteins used in this study is provided in Table 2.

**Table 2:** List of inhibitors, additives and recombinant proteins

Reagent	Source	Identifier
**Inhibitors**		
Dynasore	SCBT	sc-202592
Mitmab	Tocris	4224
MβCD	Sigma	C4555
Filipin	Sigma	SAE0087
Nystatin	Sigma	N6803
U18666A	SCBT	sc-203306
Amitriptyline	Sigma	A8404
GW4869	SCBT	sc-218578
Cytochalasin D	SCBT	sc-201442
Genistein	SCBT	sc-3515
Nocodazole	Sigma	487929
Brefildin A	SCBT	sc-200861
Golgicide A	SCBT	sc-215103
EIPA	SCBT	sc-202458
ML141	SCBT	sc-362768
NSC 23766	SCBT	sc-204823
CT04	Cytoskeleton Inc.	CT04
CAS 879127-07-8	SCBT	sc-203934
Calphostin C	Sigma	C6303
Blebbistatin	SCBT	sc-203532B
Wiskostatin	SCBT	sc-204399
Bafilomycin A1	SCBT	sc-201550A
YM201636	SCBT	sc-204193
LY 294002	Sigma	440202
Vacuolin-1	Sigma	673000
TD139	Cayman Chemical	28400
**Additives/ and recombinant proteins**		
GCDCA	Sigma	G0759
Cholesterol	Sigma	C4951
His-tagged recombinant ALIX	Fitzgerald	80R-1259
His-tagged recombinant TSG101	Fitzgerald	80R-1296
Recombinant gal-3	Biolegend	599706
Cholera Toxin B subunit	Sigma	C9903

### Preparation of J2 HIE monolayers

Human jejunal HIE was established from tissue from a patient undergoing bariatric surgery. The study protocol was approved by the Baylor College of Medicine Institutional Review Board. Preparation of HIE cultures used in the current study were described previously^2,3^. Briefly, a permissive, jejunal secretor positive (J2) HIE line was used for almost all the experiments described in this work. An isogenic, genetically modified J2Fut2 knockout HIE that lacks the FUT2 enzyme, as previously described^4^ was used in select membrane wounding studies. To prepare monolayers, 3D cultures of HIE were dissociated by trypsinization followed by gentle pipetting and passing the cells through a 40 μm cell strainer. Cells were pelleted and resuspended in a proliferation medium [CMGF(+) containing the ROCK inhibitor Y-27632 (10 μM, Sigma)], and seeded in a 96-well plate (100,000 cells per well). After 24 h of cell growth, differentiation medium was added to the cells and the cells were allowed to differentiate for 5 days with intermittent medium change.

### HuNoV infection of J2 HIE monolayers

HuNoV infection was performed with the J2 HIEs without the addition of bile/bile acids^2,3^. Briefly, J2 HIEs were incubated with 9 × 10^5^ GEs of GII.4 stool filtrate in CMGF(-) for 1 h at 37 ^o^C. The monolayers were washed twice with CMGF(-) to remove unattached viruses. One plate of infected J2 HIEs was frozen at this time point (as binding reference for 1 h) and the other plate of infected J2 HIEs were incubated for 24 h in the differentiation medium followed by Reverse Transcriptase Quantitative Polymerase Chain Reaction (RT-qPCR) to quantitate HuNoV replication.

### Toxicity assessment in HIEs

Pharmacological inhibitors were tested for toxicity in HIEs before testing their effect on HuNoV replication. The HIEs were incubated with the inhibitors for 24 h at 37 ^o^C and the supernatant was subjected to LDH-Glo™ cytotoxicity assay (Promega) according to the manufacturer’s instructions. HIE lysate was used as positive control and untreated supernatant was used as negative control and Table S1 reports toxicity results for all inhibitors.

### Infection assays in the presence of VLPs or inhibitors

The effect of VLPs or pharmacological inhibitors on GII.4 entry and infection were studied by pretreating the J2 HIE monolayers with them 1 h prior to infection. Infection was carried out in the presence of inhibitor for 1 h at 37 ^o^C. After washing and unbound virus removal, fresh media containing VLPs or inhibitors was added to the infected monolayers and incubated for 24 h until the cells were harvested for RNA isolation.

### RNA extraction and RT-PCR

Total RNA was extracted from each well using the KingFisher Flex Purification System and MagMAX™ Pathogen RNA/DNA Kit. For RT-qPCR, the primer pair COG2R /QNIF2d and probe QNIFS were used for GII.4 with qScript XLT One-Step RT-qPCR ToughMix reagent with ROX reference dye. Reactions were performed on an Applied Biosystems StepOnePlus thermocycler using the following conditions: 50 °C (15 min), 95 °C (5 min), followed by 40 cycles of 95 °C (15 s) and 60 °C (35 s). A standard curve based on a recombinant HuNoV HoV RNA transcript was used to quantitate viral GEs in RNA samples^2^.

### Western blot analysis

Samples were boiled with 4X sample buffer supplemented with *β*-mercaptoethanol at 95 °C for 10 min and subjected to SDS-PAGE for resolving the proteins. The proteins were transferred to nitrocellulose membrane and Western blot analysis was carried out using specific antibodies against target proteins. Villin and GAPDH were used as cell control which were detected using 1:1000 dilution of mouse anti-villin (#sc-373997, Santa Cruz Biotechnology) and rabbit anti-GAPDH (Cat #10494-1-AP, Proteintech) antibodies.

### Dot-blot analysis

Dot-blot analysis was carried out by adding 10 µl of purified proteins on nitrocellulose membrane as dots. After air drying, the membrane was blocked overnight at 4 ^o^C. Blocked membranes were probed with target proteins and their corresponding antibodies to detect binding.

### Immunofluorescence staining and imaging of J2 HIE monolayers

J2 HIE monolayers were either grown in 10-well glass-bottom culture slides (#543979, Greiner Bio-One) or 8-well 15 µ-slides (#80826, Ibidi) for imaging. J2 HIEs were differentiated for 5 days and infected with 1 × 10^6^ GEs GII.4. For studies using VLPs, the cells were treated with 1 × 10^12^ particles of VLPs for either 10 min or 1 h. The cells were fixed with 4% paraformaldehyde (PFA) for 20 min at room temperature, blocking with 5% BSA in 0.1% Triton X-100 (for permeabilization) in PBS for 30 min at room temperature. The cells were incubated overnight at 4 °C with primary antibodies. All the subsequent steps were performed in PBS + 0.1% Triton X-100. HuNoV capsid protein (VP1) was detected using guinea pig anti-HuNoV polyclonal Ab (pAb), 1:1000 dilution. Gal-3, ALIX, TSG101, LAMP-1, Rab11, Rab14, Flotilin-1, CD44 and glycosylphosphatidylinositol-anchored protein (GPI-AP) were detected using 1:200 dilution of rat anti-gal-3 (#125402, Biolegend), rabbit anti-ALIX (#12422-1-AP, Proteintech), anti-TSG101 (#14497-1-AP, Proteintech), rabbit anti-LAMP-1 (#9091, Cell Signaling Technologies), rabbit Rab11 (#5589, Cell Signaling Technologies) and rabbit Rab14 (#A12752, ABclonal), mouse anti-Flotilin-1 (# 610821, BD Biosciences), rabbit anti-CD44 (#15675-1-AP, Proteintech), and rabbit anti-GPI-AP (#10104-1-AP, Proteintech) antibodies, respectively. Ceramide detection was carried out as described previously^6^ using a specific rabbit anti-ceramide pAb^28,71,72^. After washing (3 times, 10 min each), the cells were incubated with 1:500 dilution of goat anti-rat 549 (#612-142-120, Rockland), donkey anti-mouse 549 (#610-742-124, Rockland), donkey anti-rabbit 649 (#611-743-127, Rockland), and anti-guinea pig 488 secondary antibodies (#606-141-129, Rockland), to visualize the viral and cellular proteins. Actin staining was done using 1:500 dilution of Alexa-647 Phalloidin (#A22287, ThermoFisher Scientific). The cells were washed three times, and nuclei were stained with 4, 6-diamidino-2-phenylindole (DAPI) (300 nM) for 5 min at room temperature followed by subsequent Z-stack images captured using a Nikon A1 confocal microscope.

The effect of inhibitors on J2 HIEs was tested by incubating the cells with pharmacological inhibitors for 24 h and fixing the cells with 4% PFA for 20 min (for actin) or methanol for 5 min (for tubulin) at room temperature. Actin was stained using Alexa-647 Phalloidin and tubulin was stained using mouse anti-tubulin antibody (#T8203, Sigma) followed by using goat anti-mouse 594 antibody(#610-742-124, Rockland) as a secondary antibody. The images were captured either by Nikon A1 confocal microscope using NIS-Elements Viewer 4.20, GE Healthcare DeltaVision Deconvolution Microscope using softWoRx-software Acquire Version: 7.2.1 or by using a ZEISS Laser Scanning Microscope LSM 980 confocal microscope using ZEISS ZEN 3.5 (blue edition) software. The images were further processed using ImageJ2/FIJI.

### Specificity testing of commercial antibodies against GII.4 VLP

Antibodies (gal-3, ALIX, LAMP-1, Rab11, and Rab14) were incubated with 5 µg of GII.4 VLPs for 1 h at room temperature. An antibody pull-down was performed by adding 25 µl of PBS-washed protein A/G magnetic beads (#88802, ThermoFisher Scientific) to the mixture and incubating the mixture for 30 min at room temperature. The beads were collected by placing tubes on a magnetic stand and the unbound proteins present in the supernatant were collected for analysis. The pelleted beads were washed with PBS (5 times) and the bound proteins were analyzed after adding 100 µl SDS-PAGE buffer and heating the beads at 95 ^o^C for 5 min (pull-down fraction). The supernatant and pull-down fractions were assessed for the presence of VLPs by SDS-PAGE, followed by Western blot analysis for the capsid VP1 protein (Fig. S6a).

### Blocking assay to block GII.4-protein interaction using antibodies

To test the blocking activity of anti-gal-3 and anti-ALIX antibodies in blocking GII.4 Syd VLP interactions with gal-3 and ALIX, antibody-blocking assays were carried out using anti-gal-3 and anti-ALIX antibodies. GII.4 Syd VLPs (2 µg) were incubated with recombinant His-tagged gal-3 and ALIX with or without anti-gal-3 (left) and anti-ALIX (right) antibodies (2 and 10 µg). A pull-down assay was carried out using Ni-NTA beads and GII.4 VP1 was detected using guinea pig (Gp) Syd-pAb (Fig. S6b).

### Epifluorescence microscopy for measurement of endocytosis

Differentiated J2 HIE monolayers (incubated with virus/VLPs for 10 min; bafilomycin for 1 h) were incubated with 50 nM LysoTracker (ThermoFisher Scientific) for 10 min. The cells were washed twice with CMGF(-) and LysoTracker staining of acidic compartments in treated and untreated J2 HIEs (mock) was observed by epifluorescence microscopy using Olympus cellSens Standard Version 2.3 software. Endocytosis measurements were carried out using FM1-43FX (ThermoFisher Scientific). HIE monolayers were treated media containing 10 μg/mL of FM1-43FX with or without VLPs for 10 min at 37 °C. Endocytosis was stopped by washing with prechilled PBS and the cells were fixed in 4% PFA for 20 min and nuclei were stained with 300 nM DAPI for 5 min at room temperature. Quantitation of the fluorescence was done using J-image. Briefly, every experiment was repeated at least three times with 3–4 images analyzed per condition in each experiment. Identical elliptical regions-of-interests (ROIs) were drawn per field and mean fluorescence intensity from these ROIs was measured.

### Dil-LDL assay

Differentiated J2 HIE monolayers were treated with dynamin inhibitors (40 µM of dynasore and 100 µM mitmab) or dimethyl sulfoxide (DMSO) for 1 h at 37 ^o^C. Dil-LDL was added to the cells (5 µl/well) in the presence of inhibitor and the cells were further incubated for 3 h at 37 ^o^C. The cells were washed three times, fixed with 4% PFA for 20 min. Dil-LDL uptake was measured using epifluorescence microscopy.

### Time lapse microscopy

Time lapse microscopy was carried out by adding fluorescently labeled VLPs with FM1-43FX to J2 HIE monolayers and subsequent Z-stack images were captured (every 2.5 min for a period of 1 h at 37 ^o^C) using a GE Healthcare GE Healthcare DeltaVision Deconvolution Microscope. Image acquisition was done using softWoRx-Acquire Version: 7.2.1 software. The image stack was then converted into a movie using ImageJ AVI pluggin.

### Thin section electron microscopy

J2 HIE monolayers were incubated with VLPs for 1 h at 37 ^o^C. The cells were washed twice with PBS and VLP-treated and untreated control cells were trypsinized and fixed in 2% paraformaldehyde–3% phosphate-buffered glutaraldehyde. Thin sections were prepared and electron microscopy was carried out using a FEI Tecnai Spirit transmission electron microscope equipped with a 4 K Eagle digital camera^73^.

### Affinity measurements using BLI

BLI studies were carried out using an Octet RED96 instrument (ForteBio). Biotinylation of ALIX, TSG101 and gal-3 was carried out using EZ-Link NHC-LC-LC-biotin following the manufacturer’s instructions. The biotinylated proteins, bTSG101 (0.5 μg/ml), bALIX (1 μg/ml), bgal-3 (1 μg/ml) and were loaded onto streptavidin biosensors (18-5019, ForteBio) in the running buffer (20 mM HEPES, 150 mM NaCl, 0.005% surfactant Tween 20, and 2 mg/ml BSA, pH 7.8) for 600 s. Affinity measurements were carried out by passing two-fold serial dilutions of recombinant GII.4 S (280-70 μM) and P domains (144-36 μM) over the captured bALIX, bTSG and bgal-3 and allowing both association and dissociation for 900 s using ForteBio data acquisition software Version 7.1.0.100. The binding data were fitted using the ForteBio data analysis software Version 7.1.0.38 (2:1 model) by subtracting buffer blanks to calculate the binding affinity of GII.4 S and P domains with biotinylated cellular proteins (ALIX, TSG, and gal-3).

### Elisa

Direct binding of ALIX with GII.4 domains (S and P) and VLP was determined by ELISA. ALIX (5 μg/ml)-coated and blocked plates were incubated with equimolar concentrations of GII.4 VLP, S and P domain for 2 h at room temperature. The plate was washed three times with PBST (PBS with 0.05% Tween 20), and PBS followed by probing the interaction with guinea pig anti-HuNoV polyclonal Ab, 1:5000 dilution in 1% blocking buffer. Horseradish peroxidase-labeled goat anti-guinea pig secondary antibody (1:5000) was used to detect the binding using tetramethylbenzidine (TMB) substrate. Color development was quenched using phosphoric acid and absorbance was measured at 450 nm.

### Generation of GII.4 ΔS-domain

The GII.4 ΔS-domain construct containing the “AATAL” mutation at the putative ALIX binding site was generated using the NEBaseChanger software and Q5 site-Directed Mutagenesis Kit. The sequence of the primers used was TGCGCTGCGTGCGAACAATGCT and GTCGCCGCCATTGCGATCAGTTTGATGGTC (Integrated DNA Technologies). The mutant construct was then transformed and cultured in Novablue cells made competent using a Mix & Go transformation kit (Zymo Research). Cells were then cultured in 50 ml Luria Broth media, containing 50 µg/ml ampicillin, and purified using Qiagen Miniprep kit (Qiagen). Purified DNA was then submitted to Genewiz for Sanger sequencing to confirm the successful incorporation of the AATAL mutation. His-tagged shell mutant was then expressed, purified and assessed using EM and SDS-PAGE.

### Generation of GII.4 Syd ΔVLP

To obtain mutant VLPs, the ORF1 + ORF2 + 3’ UTR nucleotide sequence of norovirus GII.4 Sydney (JX459908) was submitted to a commercial laboratory (Epoch Life Science) for modification. Nucleotides 5628–5637 of the cDNA version of the genome were changed from TTGTATACAC to GCAGCTACAG. The coded amino acids 182–185 of VP1 were thus mutated from “LYTPL” to “AATAL”. The laboratory synthesized the gene and after verification of the sequence, the insert was cloned into the bacmid pFastbac1 vector. The Protein and Monoclonal Antibody Production Core at Baylor College of Medicine recombinantly expressed VLPs by infecting High Five™ (#B85502, ThermoFisher Scientific) insect cells with the bacmid construct. The VLPs were purified by passing through a 30% sucrose cushion followed by a cesium chloride gradient and dialysis in sodium chloride/sodium phosphate buffer as previously described^74^.

### shRNA-mediated knockdown of the *PDCD6IP* gene

A microRNA-adapted shRNA technology based on miR-30 was used to knockdown gene expression in the J2 HIE line. Commercially available pGIPZ lentiviral constructs, comprising of shRNAs complementary to the sequence of the targeted *PDCD6IP* gene (#RHS4430-200156283, #RHS4430-200158656, #RHS4430-200283070; Dharmacon), were provided as bacterial glycerol stocks. shRNA constructs were extracted using Qiagen prep kit (Qiagen).

Lentiviral particles with the shRNAs were produced, using the third-generation lentivirus technology, by co-transfecting HEK293FT cells (#R70007, ThermoFisher Scientific) with a combination of each GIPZ-shRNA lentiviral plasmid and three packaging plasmids pMDLg/pRRE [plasmid #12251; Addgene], envelope plasmid pMD2.G [plasmid #12259; Addgene], and pRSV-Rev [plasmid #12253; Addgene]) at a ratio of 3.5:2:1:1, respectively, using polyethyleneimine HCl Max molecular weight (MW) 40,000 (Polysciences). The culture supernatant was harvested 72 h, passed through a 0.45-μm filter, concentrated by using LentiX-concentrator (TaKaRa-Clontech) according to the manufacturer’s protocol, and suspended in a high Wnt-3A proliferation medium supplemented with 10 μM CHIR99021 and 10 μM Rho-associated protein kinase (ROCK) inhibitor Y-27632, and stored at −80 °C in 50 μl aliquots until further use.

A cell suspension was prepared from three-dimensional (3D) undifferentiated J2 HIEs cultivated as previously described. After trypsinization and pelleting of the cells at 300 × *g*, the resulting cell pellet was suspended at a concentration of 3 × 10^5^ cells per ml in high Wnt-3A proliferation medium supplemented with 10 μM CHIR99021 and 10 μM Y-27632. 8 μg/ml Polybrene and 50 μl of concentrated shRNA lentivirus were added to 1 × 10^5^ cells, the mixture was plated in one well of a 48-well plate and centrifuged for 1 h at 600 × *g* at room temperature. After spinoculation, the plate was incubated for 4 h at 37 °C under 5% CO_2_ atmosphere to enhance transduction. The lentivirus solution was removed by centrifugation, the cells were washed once with CMGF(-) medium, centrifuged again, embedded in 30 μl Matrigel plug, and incubated at 37 °C and 5% CO_2_ in the presence of high Wnt-3A medium with CHIR99021 and Y-27632 for recovery. Five days post-transduction, the cells were treated with puromycin(2 μg/ml) until mock-treated cells were completely dead. Single-cell clones were isolated by serial dilution of cells in 96-well plates for shRNA-transduced HIEs, and deletion of the gene was confirmed by sequencing of genomic DNA from each single cell clone using primers ALIX-F and ALIX-R that amplify the portion of the shRNA targeted *PDCD6IP* gene.

### Wounding assays

Differentiated J2 or J2FUT2KO HIEs were incubated with a mixture of VLP and PI (5 µg/ml) for 10 min at 37 ^o^C. The cells were washed twice with CMGF(-) and fixed with 4% PFA for 20 min. After incubation, the cells were washed with PBS and counterstained with DAPI for 5 min. PI/DAPI colocalization was measured to determine VLP-induced wounding.

### Statistical analysis

Each experiment was performed three or more times independently with two or more HIE replicates (represented by n). For infection assays, two HIE replicates for 1 h time point and three independent HIE replicates for the 24 h time point were used. RT-qPCR assays were carried out using two or more technical replicates for each HIE replicate depending on the treatment of cells. Samples with RNA levels below the limit of detection of the RT-qPCR assay were assigned a value that was one-half the limit of detection of the assay. All statistical analyses were performed on GraphPad Prism (Version 8.4.3) (GraphPad Software) using geometric mean values. Comparison between untreated and treated groups at 24 h was performed using one-way ANOVA using data from independent HIE replicates of one independent experiment, with statistical significance (*P* value) determined using Dunnett’s multiple comparisons test. For imaging experiments, statistical significance was calculated either by one-way ANOVA, Dunnett’s multiple comparisons test (when comparing with control) or using two-way ANOVA with Tukey’s or Šídák’s multiple comparisons test (when comparing within groups). A *P* value above 0.05 was considered non-significant.

### Reporting summary

Further information on research design is available in the Nature Portfolio Reporting Summary linked to this article.

## Supplementary information

Supplementary Information

Reporting Summary

## Data Availability

The authors declare that all data supporting the findings of this study are available within the paper and in the Supplementary Information files. Raw data is available as Source Data file. Source data are provided with this paper.

## Source data

Source Data
