# Supplementary material for: CLIC and membrane wound repair pathways enable pandemic norovirus entry and infection
Source: Nat Commun. 2023 Feb 28;14:1148. doi: 10.1038/s41467-023-36398-z (PMC9974061; doi:10.1038/s41467-023-36398-z)
Supplement: Supplementary file 1 — Supplementary Information [file 41467_2023_36398_MOESM1_ESM.pdf]

Supplemental information

CLIC and Membrane Wound Repair Pathways Enable Pandemic Norovirus Entry and Infection

B. Vijayalakshmi Ayyar, Khalil Ettayebi, Wilhelm Salmen, Umesh C. Karandikar, Frederick H. Neill, Victoria R. Tenge, Sue E. Crawford, Erhard Bieberich, B. V. Venkataram Prasad, Robert L. Atmar, Mary K. Estes

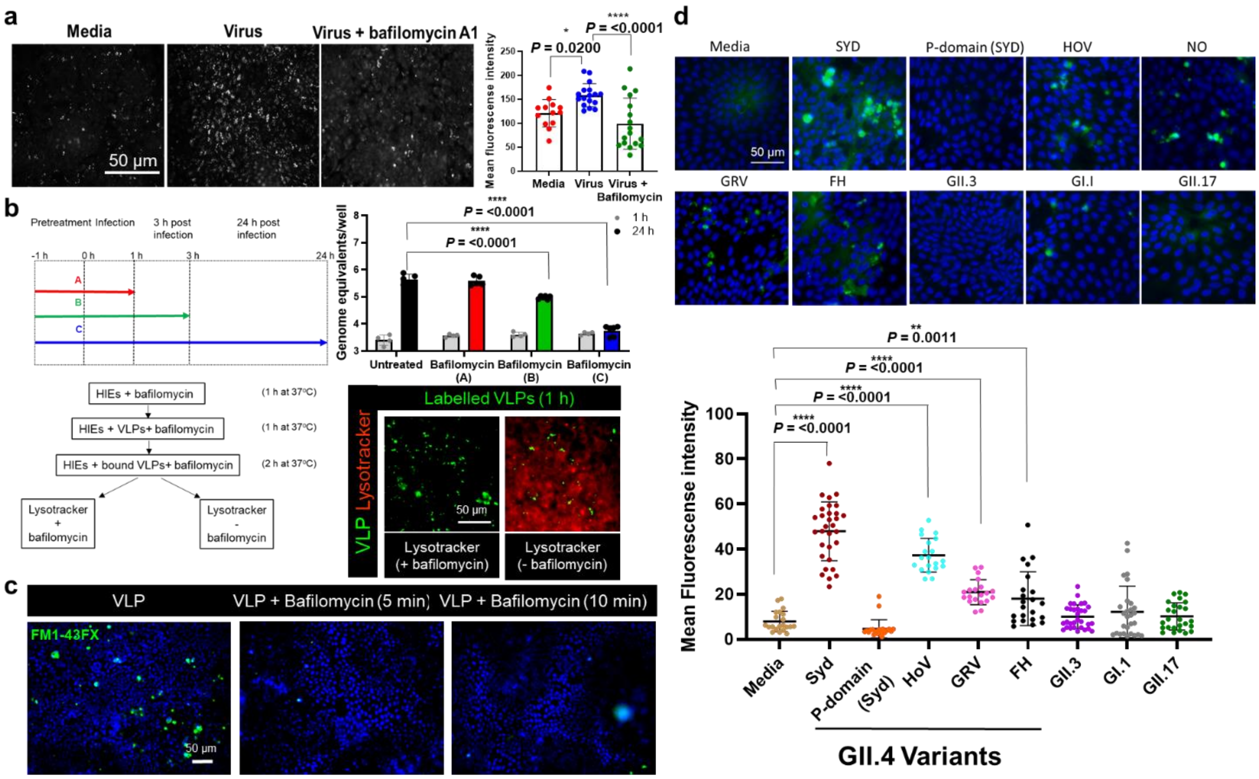

Supplementary Figure 1. GII.4 induces strain-specific endocytosis dependent upon V-ATPase regulated endosomal acidification

a. Bafilomycin A1 (1 h pretreatment) reduces GII.4-induced LysoTracker positive compartments as observed by epifluorescence microscopy. Right panel: Mean fluorescence intensity of LysoTracker positive compartments in media (red dots, ROI= 13), virus (blue dots, ROI=17) and virus + bafilomycin (green dots, ROI=17) using n=3 HIE replicates.

b. Left panel: Schematic of experimental design. (Line A, red bar) represents bafilomycin A1 treatment 1 h before virus addition and 1 h during inoculation; (Line B, green bar) represents bafilomycin A1 treatment 1 h before virus addition followed by 1 h during inoculation and leaving the inhibitor on the cells for another 2 h; (Line C, blue bar) represents the presence of inhibitor 1 h before infection followed by 1 h during inoculation and throughout 24 h. Right panel: Effect of bafilomycin A1 (12.5 nM treatment time on GII.4 replication was studied by quantifying GII.4 replication at 1 h (grey) and 24 h (black, red, green, blue) using n=2 independent HIE replicates for the 1 h and n= 3 independent HIE replicates for 24 h, with 2 technical replicates/sample. Bottom Left panel shows the schematic of the experiment. Bottom right panel: Bafilomycin A1-induced V-ATPase inhibition is reversible as observed

with LysoTracker positive compartments (red) induced by membrane-bound labeled GII.4 VLPs (green) by epifluorescence microscopy in the absence and with washout of bafilomycin A1.

c. GII.4 VLP-induced endocytosis [FM1-43FX uptake (green)] is reduced on inhibiting V-ATPase with bafiolmycin treatment.

d. Endocytosis (FM1-43FX uptake) after inoculation with GII.4 variants [Sydney/2012 strain (SYD), Houston/2002 strain (HOV), New Orleans/2009 strain (NO), Grimsby/1995 strain (GRV) and Farmington Hills/2002 strain (FH)], GII.3, GII.17 and GI.1 along with a recombinant GII.4 Syd P-domain. Lower Panel: Quantitation of endocytic vesicles (green) was carried out using n=2 HIE replicates/ condition from various ROIs [media, tan (ROI=21); Syd, red (ROI=31); P-domain (Syd), orange (ROI=23); HoV, blue (ROI=20); GRV, pink (ROI=21); FH, black (ROI=22); GII.3, purple (ROI=31); GI.1, grey (ROI=32); and GII.17, green (ROI=26)]

All the experiments were repeated independently 3 times with similar results. In figures a, b and d, error bars represent mean ± SD with significance (P values) was calculated using one-way ANOVA, Dunnett's multiple comparisons test. Source data are provided as a Source Data file.

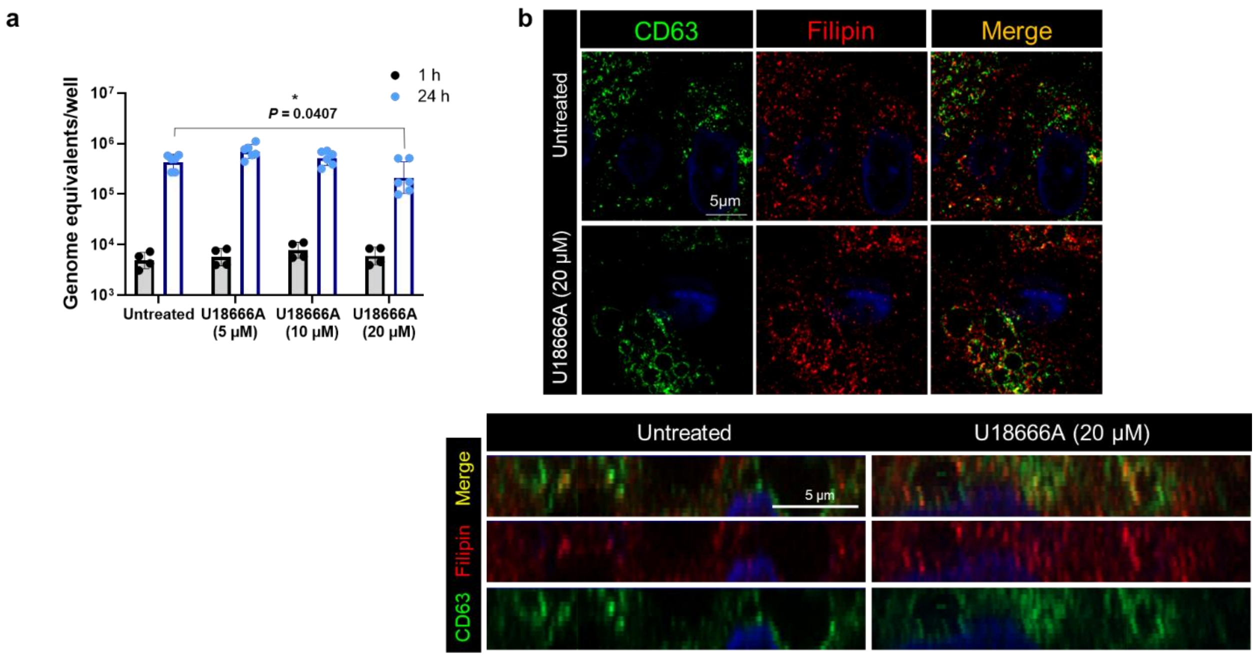

Supplementary Figure 2. Membrane cholesterol transport is critical for GII.4 entry

a. Inhibition of intracellular cholesterol transport using U18666A, a Niemann-Pick C1 inhibitor, reduces GII.4 replication. HIEs were incubated with different concentrations of U18666A at 37°C. GII.4 replication was quantified at 1 h (black) and 24 h (blue) in the presence/ absence of U18666A using n=2 independent HIE replicates for the 1 h and n= 3 independent HIE replicates for 24 h, with 2 technical replicates/sample. Error bars represent mean ± SD with significance (*P* values) relative to untreated at 24 h was calculated using one-way ANOVA, Dunnett's multiple comparisons test. The experiments were repeated independently three times with similar results. Source data are provided as a Source Data file. b. U18666A treatment of HIEs results in intracellular accumulation of CD63 and cholesterol detected using murine anti-CD63 mAb (green) and filipin (red), respectively. Lower panel shows orthogonal views of the corresponding image. The experiment was repeated 3 times with similar results.

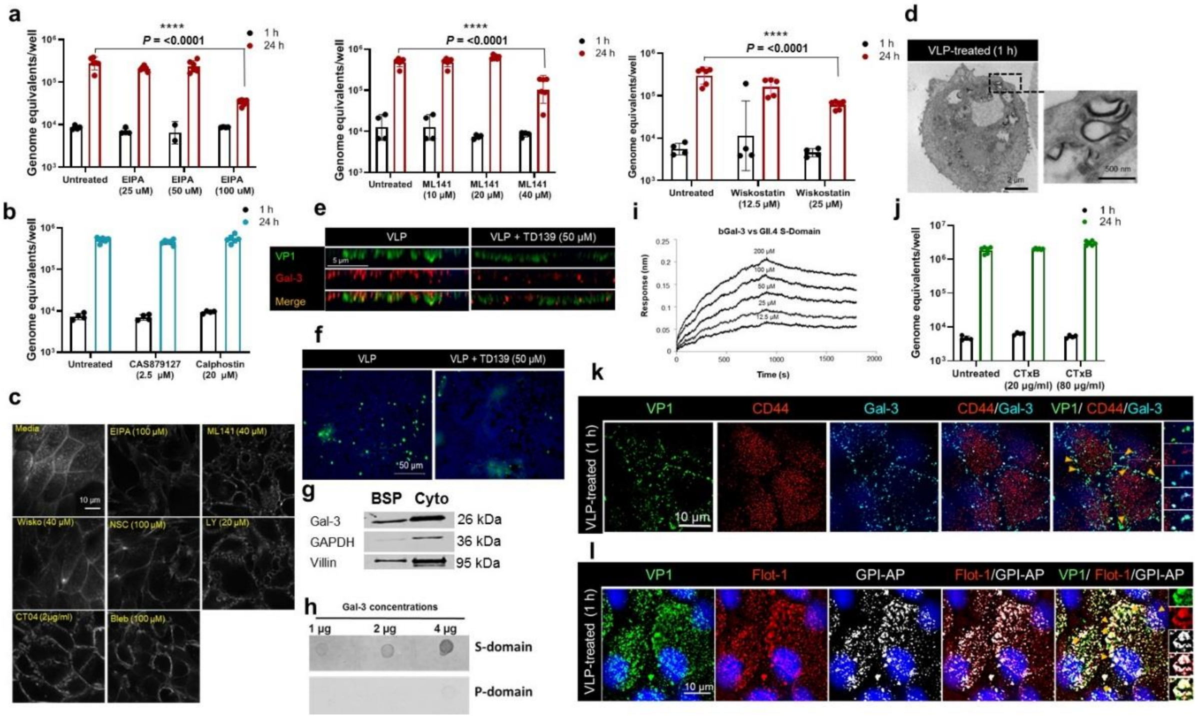

Supplementary Figure 3. GII.4 entry is via CLIC-mediated endocytosis mediated by a Cdc42-regulated actin and gal-3 interactions.

a. Cdc42 inhibitors reduce GII.4 replication in a dose-dependent manner. Left panel: EIPA; Center panel: ML141; Right panel: Wiskostatin. GII.4 replication was quantified at 1 h (black) and 24 h (red) in the presence/ absence of inhibitor using n= 2 independent HIE replicates for the 1 h and n= 3 independent HIE replicates for 24 h, with 2 technical replicates/sample.

b. Effect of EGFR (CAS879127) and PKC (calphostin) inhibitors on GII.4 replication. 1 h (black) and 24 h (blue) in the presence/ absence of inhibitor using n= 2 independent HIE replicates for the 1 h and n= 3 independent HIE replicates for 24 h, with 2 technical replicates/sample.

c. Efficacy of inhibitors in HIEs was confirmed using actin staining (white) followed by confocal microscopy. HIEs were incubated with inhibitors for 24 h at 37°C followed by actin staining using Alexa Fluor™ 647 Phalloidin.

d. Electron micrographs (EM) showing GII.4 VLPs are present in both crescent-shaped structures with CLIC morphology and also in some vacuolar structures (inset). EM was done once using 3 HIE replicates for both VLP-treated and mock-treated cells.

e. Gal-3 (red)-VP1 (green) colocalization in the presence of the gal-3 inhibitor TD139 compared to untreated HIEs.

f. Gal-3 inhibition with 50 µM gal-3 inhibitor TD139 (added to HIEs 30 min prior to incubation with GII.4 VLPs) reduces GII.4 endocytosis (FM1-43FX uptake).

g. Localization of gal-3 in the biotinylated surface protein (BSP) fraction and cytoplasmic (Cyto) fraction was evaluated by Western blotting. The membrane protein villin and cytoplasmic protein GAPDH were used as controls.

h. Dot blot analysis of gal-3 with individual S and P domains of GII.4 Syd showing that gal-3 binds the GII.4 Syd S domain. Recombinant gal-3 (1 µg, 2 µg and 4 µg) was coated on a nitrocellulose membrane that was blocked and incubated with 5 µg of the different GII.4 capsid domains followed by detection with Gp Syd-pAb.

i. Bio-Layer Interferometry (BLI) showing that GII.4 S-domain binds gal-3 with micromolar affinity.

j. GII.4 replication in the presence of CTxB at 1 h (black) and 24 h (green) using n=2 independent HIE replicates for the 1 h and n= 3 independent HIE replicates for 24 h, with 2 technical replicates/sample.

k. GII.4 VP1 colocalization with gal-3 and CD44 in VLP-treated cells. VP1 (green), CD44 (red), and gal-3 (blue) were detected using GpSyd-pAb, anti-gal-3 and anti-CD44 antibody (# 15675-1-AP, Proteintech) (n=2 HIE replicates). Yellow represent colocalization. Inset: GII.4-gal-3-CD44 colocalization.

l. GII.4 VP1 colocalization with lipid raft marker flotillin-1 (Flot-1) and glycosylphosphatidylinositol-anchored protein (GPI-AP). VP1 (green), Flot-1 (red) and GPI-AP (white) were detected using GpSyd-pAb, anti-Flot-1 (# 610821, BD Biosciences) and anti-GPI-AP antibody (#10104-1-AP, Proteintech) (n=2 HIE replicates) Yellow arrows represent colocalization. Inset: GII.4-Flot-1-GPI-AP colocalization.

All the experiments were repeated independently 3 times with similar results. In figure a, b and j, error bars represent mean ± SD with significance (P values) calculated using one-way ANOVA, Dunnett’s multiple comparisons test. Source data are provided as a Source Data file.

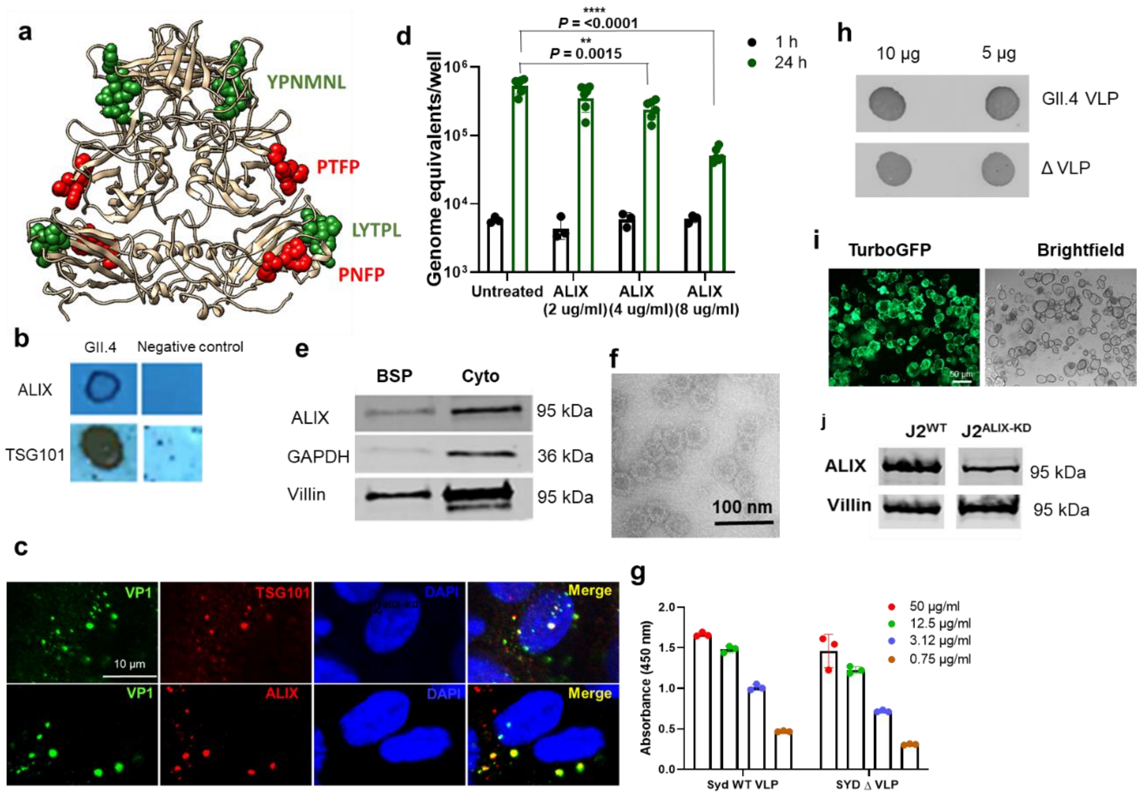

Supplementary Figure 4. GII.4 interacts with ESCRT proteins which regulate its entry and replication in HIEs

a. Putative ALIX (green) and TSG101 (red) binding motifs shown as sphere model and highlighted in GII.4 HOV VP1 dimer [7K6V (http://doi.org/10.2210/pdb7K6V/pdb)]. Image was prepared using chimera software.

b. Direct binding dot blot assay showing GII.4 binding with immobilized ALIX and TSG101. 10% BSA was used as negative control.

c. TSG101 and ALIX colocalization with GII.4 virus at 24 h by confocal microscopy. GII.4 VP1 (green), TSG101 (red, upper panel) and ALIX (red, lower panel) were detected using Gp Syd-pAb for GII.4 VP1 and rabbit pAbs for ALIX and TSG101 (red).

d. Dose-dependent inhibition of GII.4 replication using anti-ALIX pAb. Replication was quantified at 1 h (black, n=2 independent HIE replicates) and 24 h (green, n=3 independent HIE replicates) with two technical replicates for each condition. Error bars represent mean ± SD with significance relative to control (P values) calculated using one-way ANOVA, Dunnett's multiple comparisons test.

e. Localization of ALIX on differentiated HIEs by Western blot analysis of biotinylated surface protein (BSP) and cytosolic (Cyto) protein fractions using rabbit anti-ALIX antibody. Membrane protein villin and cytoplasmic protein GAPDH were used as controls.
f. Electron micrograph of negative stains (using 2% uranyl acetate) showing that mutation of ALIX binding site did not interfere with VLP formation.
g. Mutating the putative ALIX binding site on the GII.4 VLP had no effect on GII.4-ALIX interactions as observed by ELISA. Error indicates mean ± SD with three replicates.
h. Dot blot assay showing ALIX interaction with immobilized GII.4 Syd VLPs and Syd Δ VLPs, probed using rabbit anti-ALIX pAb.
i. Fluorescent microscopy and bright field images showing successful generation of ALIX knock down J2 HIEs ($J2^{ALIX\text{-}KD}$) using lentiviral particles generated from the pGIPZ ALIX (gene symbol *PDCD6IP*) shRNA TurboGFP vector.
j. Western blot analysis detected knock down of ALIX in $J2^{ALIX\text{-}KD}$ HIEs compared to $J2^{WT}$ HIEs. Villin was used as a loading control.

All the experiments were repeated independently 3 times with similar results. Source data are provided as a Source Data file.

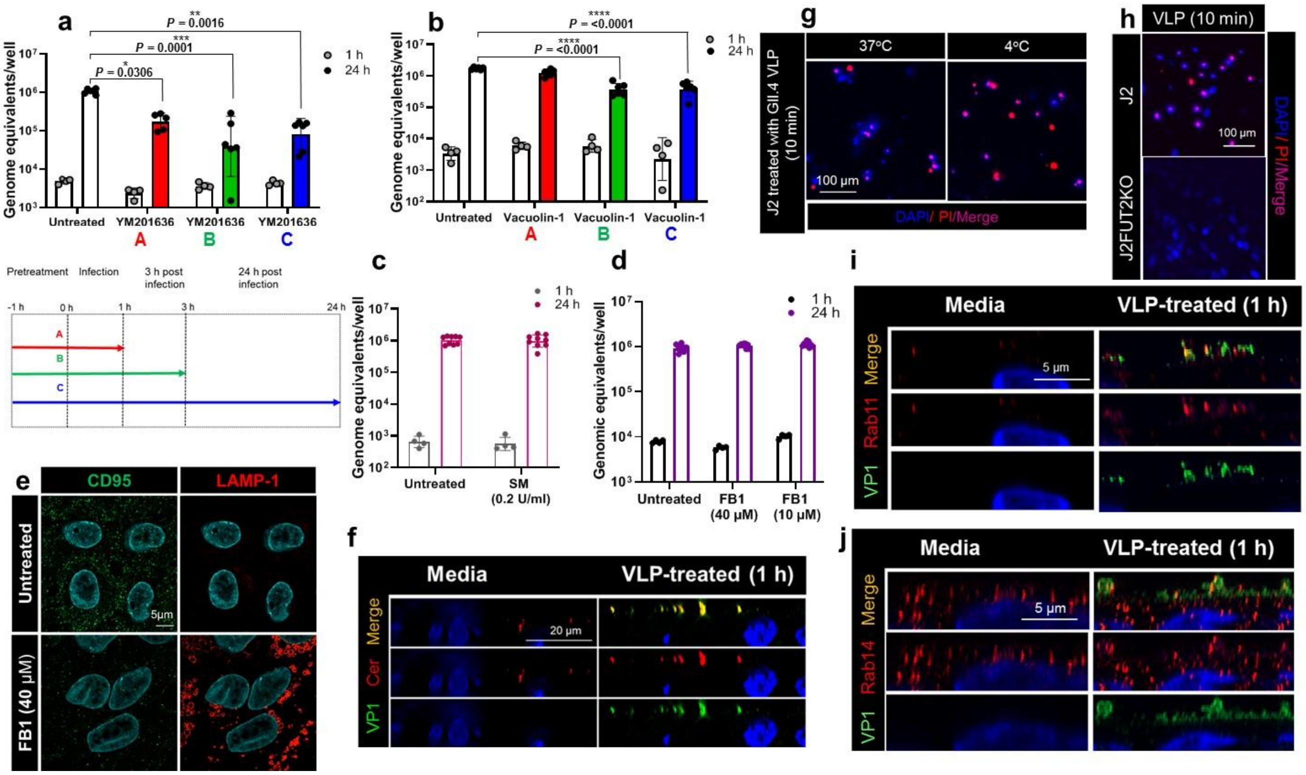

Supplementary Figure 5. GII.4 entry interferes with endo-lysosomal trafficking causing lysosomal exocytosis and surface ceramide generation, and HBGA-dependent plasma membrane wounding

a. Effect of YM201636 (PIKfyve inhibitor) on GII.4 replication (top panel). Schematic illustrating HIE incubation with inhibitor for different time periods (bottom panel): (Line A, red line or bar with black dots) represents GII.4 replication at 24 h when inhibitor was incubated 1 h before virus addition and 1h during virus incubation; (B, green line or bar with black dots) represents GII.4 replication at 24 h when inhibitor was incubated 1 h before virus addition followed by 1 h during infection and 2 h. (C, blue line or bar with black dots) represents the presence of inhibitor 1 h before infection followed by 1 h during infection and 24 h (black dots). GII.4 binding to HIEs at 1 h is represented by grey dots. Assay was performed with n=2 HIE replicates for 1 h and n=3 HIE replicates for 24 h with 2 technical replicates for each condition.
b. Effect of vacuolin-1 (lysosomal exocytosis inhibitor) treatment on GII.4 replication. A (red bar with black dots) represents GII.4 replication at 24 h when inhibitor was incubated 1 h before virus addition and 1 h during virus incubation; B, (green bar with black dots)

represents GII.4 replication at 24 h when inhibitor was incubated 1 h before virus addition followed by 1 h during infection and 2 h. C, (blue bar with black dots) represents the presence of inhibitor 1 h before infection followed by 1 h during infection and 24 h (black dots). GII.4 binding to HIEs at 1 h is represented by grey dots. Assay was performed with n=2 HIE replicates for 1 h and n=3 HIE replicates for 24 h with 2 technical replicates for each condition.

c. GII.4 replication in the presence of sphingomyelinase (SM). Bacterial SM from *Bacillus cereus* was exogenously added to HIEs prior to and during infection to catalyze surface ceramide formation and evaluate its effect on GII.4 replication. HIEs were infected with GII.4 virus and replication were quantified at 1 h (grey) and 24 h (pink). Assay was performed with n=2 HIE replicates for 1 h and n=3 HIE replicates for 24 h with 3 technical replicates for each condition.
d. GII.4 replication in the presence of ceramide synthase inhibitor, fumonisin B1 (FB1) at 1 h (black) and 24 h (purple). Assay was performed with n=2 HIE replicates for 1 h and n=3 HIE replicates for 24 h with 3 technical replicates for each condition
e. Confocal microscopy to confirm FB1 efficacy in HIEs by staining the FB1-treated and untreated cells for CD95 using Fas ligand antibody (#556372, BD biosciences) and LAMP-1 expression using specific mAbs.
f. VLP-induced apical ceramide (red) was detected using confocal microscopy. GII.4 VP1 (green) was detected using by Gp Syd-pAb and ceramide (red) was detected using anti-ceramide rabbit pAb.
g. Membrane wounding due to VLP binding was investigated using PI assay. DAPI (blue) and PI (red) visualization indicated for wounding.
h. Membrane wounding due to GII.4 VLP binding to cell surface glycans was analyzed using J2 [secretor positive, expressing plasma membrane fucosylated histoblood antigens (HBGAs)] HIEs and an isogenic J2FUT2KO (J2 with Fut2 knocked out, secretor negative lacking plasma membrane HBGAs) and the PI assay. Membrane wounding was determined using DAPI (blue) and PI (red) colocalization.
i. Role of endosomal recycling and lysosomal exocytosis in GII.4 entry into HIEs was determined by probing GII.4-Rab11 colocalization. GII.4 VP1 (green) and Rab11 (red) were detected using Gp Syd-pAb and rabbit pAb.

j. GII.4 colocalization with Rab14, a cytokinesis and recycling endosomal marker, was evaluated by confocal microscopy. GII.4 VP1 (green) was detected using by Gp Syd-pAb and Rab14 (red) was detected using rabbit pAb.

All the experiments were repeated independently 3 times with similar results. In figures a, b, c and d, error bars represent mean ± SD with significance relative to control (P values) calculated using one-way ANOVA, Dunnett's multiple comparisons test. Source data are provided as a Source Data file.

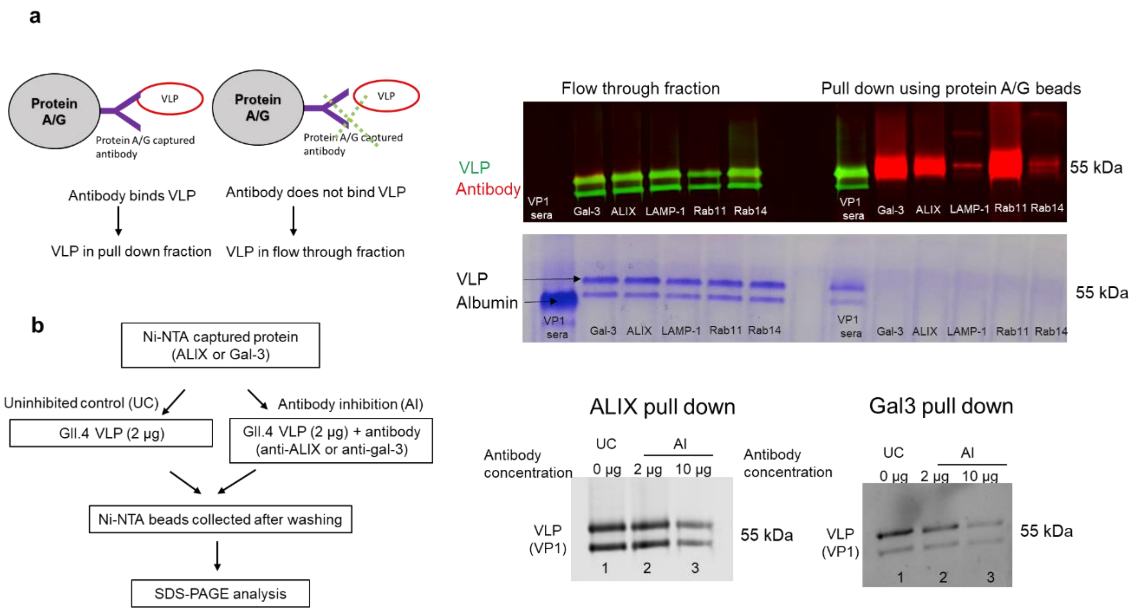

Supplementary Figure 6. Antibodies to cellular host proteins lack cross-reactivity to GII.4 VLPs but anti-Gal-3 and anti-ALIX antibodies block VLP-host protein interactions.

a. GII.4 VLPs (5 µg) in separate tubes were incubated overnight at 4°C with equimolar concentrations of rat-anti-gal-3, rabbit anti-ALIX, rabbit anti-LAMP1, rabbit anti-Rab11 and rabbit anti-Rab14 antibodies, respectively. VP1 GpSyd-pAb (10 µl) was used as a control. The antibodies were captured by adding 25 µl of PBS-washed protein A/G magnetic beads to the mixture. The flow-through fraction representing unbound material and the pull-down fraction representing the antibody-bound material were collected and analyzed by Western blots and SDS-PAGE. Left panel: schematic of the experiment. Right panel (top): Western blot analysis detecting the VLP capsid VP1 protein (left side - green, using guinea pig anti-VP1 antibody) present in the flow through fractions and protein on the beads in the pull down fraction on the beads captured with each individual antibody (right side-red, detected using a mixture of anti-rat and rabbit antibodies). The VP1 capsid protein is detected as two bands with one band being a well-known VP1 cleavage product. Bottom right panel: Coomassie blue stained SDS-PAGE gel showing that only the positive Gp Syd-pAb control that is specific to the VP1 capsid protein of VLPs and none of the other antibodies used in the pull down assay) bound GII.4 VLP.

b. A blocking assay was performed by incubating GII.4 VLP (2 µg) in separate tubes containing either His-tagged recombinant ALIX or gal-3 proteins without [uninhibited control, UC)] or

with antibodies [antibody inhibition (AI)]. 2 µg and 10 µg of anti- ALIX (left) or anti- gal-3 (right) antibodies were used for the assay. His-tagged proteins (ALIX/gal-3) were isolated from the mixture using Ni-NTA beads and the amount of VLP bound to ALIX and gal-3 (in the presence and absence of competing anti-ALIX or anti-gal-3 antibody) was determined by Western blotting using GII.4 specific Gp Syd-pAb. Left panel shows the schematic of the experiment and the bottom right panel shows Western blotting of the isolated beads.1: His-tagged ALIX/gal-3 (1 µg) + 2 µg GII.4 VLP (pull-down using Ni-NTA beads); 2: His-tagged ALIX/gal-3 (1 µg + 2 µg anti-ALIX/anti-gal-3 + 2 µg GII.4 VLP (pull-down using Ni-NTA beads) and 3: His-tagged ALIX/gal-3(1 µg + 10 µg anti-ALIX/ anti-gal-3 + 2 µg GII.4 VLP (pull-down using Ni-NTA beads).

Table S1 Toxicity Assessment of Inhibitors

Target	Inhibitor	Concentration	% Viability after treatment
Control	DMSO	1:200	100%
Dynamin	Mitmab	100 µM	83.9-89.6%
	Dynasore	40 µM	96.4-99.2%
Cholesterol	MβCD	1 mM	100%
	Filipin	4 µg/ml	91.6-92.4%
	U18666A	20 µM	100%
Microtubules	Nocodazole	20 µM	81-84.9%
RTK	Genistein	80 µM	91.7-97.8%
Actin	Cytochalasin D	5 µM	92.3-96.9%
	EIPA	100 µM	93.4-94.8%
	ML141	40 µM	100%
	NCS23766	100 µM	100%
	CT04	2 µg/ml	86.5-99.7%
	Wiskostatin	25 µM	100%
Arf1	Golgicide A	4 µM	94.3-100%
Myosin	Blebbistatin	100 µM	98.7-99.2%
PI3K	LY29402	20 µM	100%
EGFR	CAS879127	2.5 µM	95.5-100%
	Calphostin	20 µM	84.2-86.3%
V-ATPase	Bafilomycin A	50 µM	100%
PIKfyve	YM 201636	4 µM	85.9-88.8%
Lysosomal exocytosis	Vacuolin 1	10 µM	93.3-96.7%
ASM	Amitriptyline	50 µM	100%
NSM	GW4869	10 µM	81.8-84.6%
