# Supplementary material for: CLIC and membrane wound repair pathways enable pandemic norovirus entry and infection
Source: Nat Commun. 2023 Feb 28;14:1148. doi: 10.1038/s41467-023-36398-z (PMC9974061; doi:10.1038/s41467-023-36398-z)
Supplement: Supplementary file 3 — Source Data [file 41467_2023_36398_MOESM3_ESM.zip › Source data/source data blots.pptx]

## Slide 1
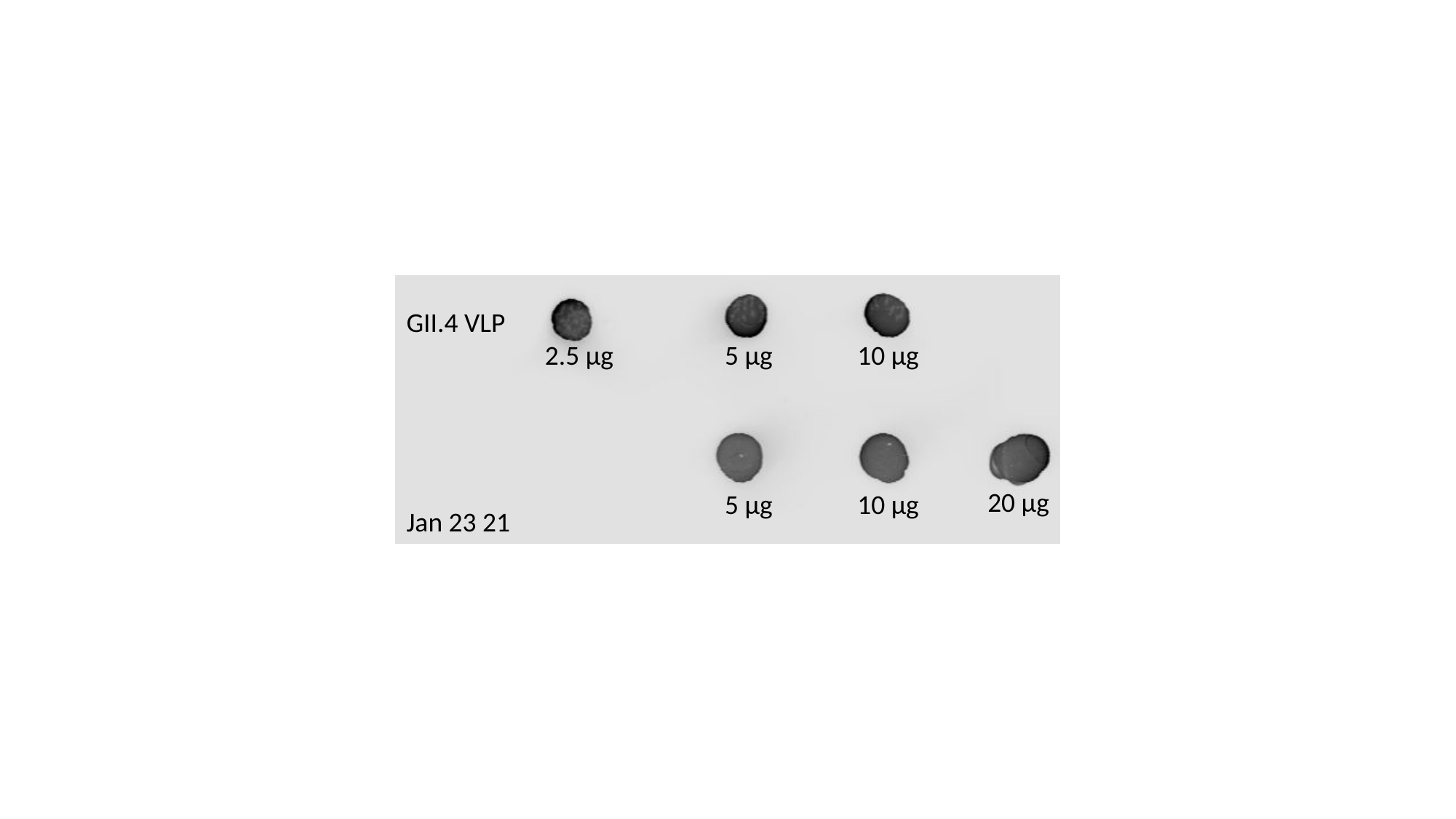

GII.4 VLP
2.5 μg
5 μg
10 μg
20 μg
5 μg
10 μg
Jan 23 21

## Slide 2
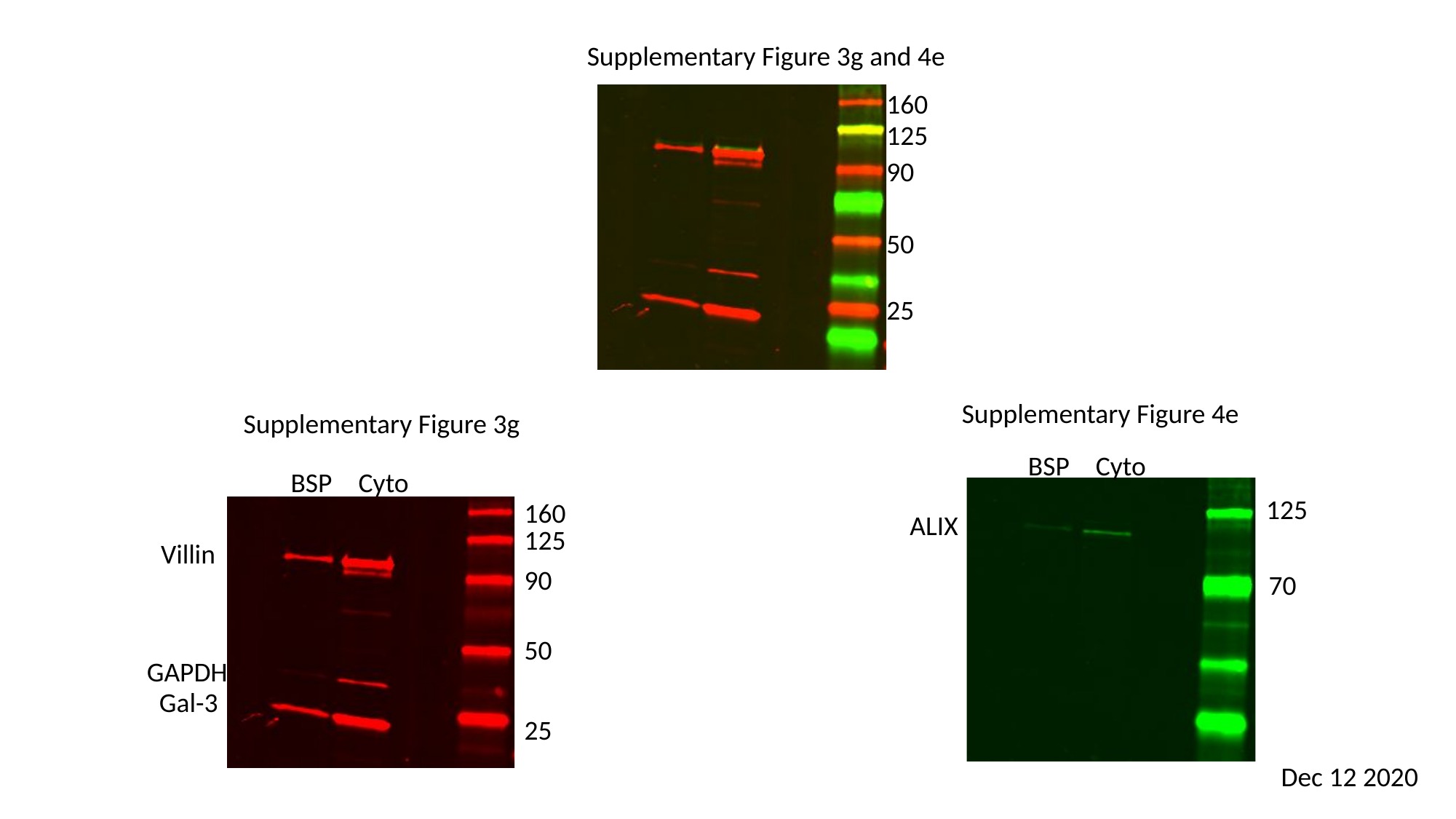

Supplementary Figure 3g and 4e
160
125
90
50
25
Supplementary Figure 4e
BSP
Cyto
125
ALIX
70
Supplementary Figure 3g
BSP
Cyto
160
125
Villin
90
50
GAPDH
Gal-3
25
Dec 12 2020

## Slide 3
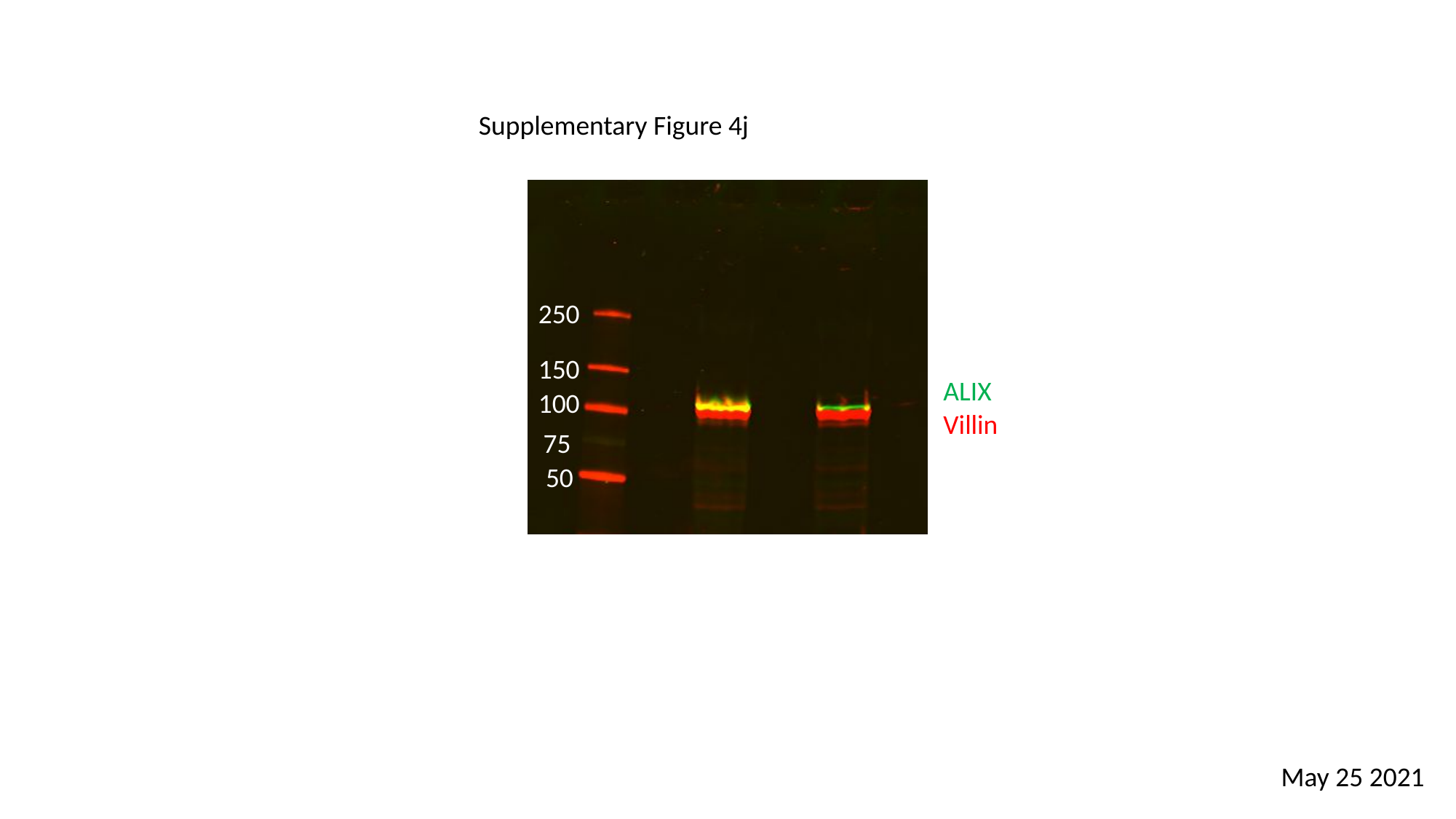

Supplementary Figure 4j
250
150
ALIX
100
Villin
75
50
May 25 2021
